# Crystal structures of an imidazo[1,5-*a*]pyridinium-based ligand and its (C_13_H_12_N_3_)_2_[CdI_4_] hybrid salt

**DOI:** 10.1107/S2056989019009964

**Published:** 2019-07-19

**Authors:** Olga Yu. Vassilyeva, Elena A. Buvaylo, Vladimir N. Kokozay, Brian W. Skelton, Alexandre N. Sobolev

**Affiliations:** a Department of Chemistry, Taras Shevchenko National University of Kyiv, 64/13 Volodymyrska Street, Kyiv 01601, Ukraine; b School of Molecular Sciences, M310, University of Western Australia, Perth, WA 6009, Australia

**Keywords:** organic–inorganic hybrid, tetra­halometallate, crystal structure, hydrogen-bonding inter­actions, π–π stacking

## Abstract

The monocation product of the oxidative condensation–cyclization between two mol­ecules of pyridine-2-carbaldehyde and one mol­ecule of CH_3_NH_2_·HCl in methanol, 2-methyl-3-(pyridin-2-yl)imidazo[1,5-*a*]pyridinium, was isolated in the presence of metal ions as bis­[2-methyl-3-(pyridin-2-yl)imidazo[1,5-*a*]pyridin-2-ium] tetra­iodo­cadmate, (C_13_H_12_N_3_)_2_[CdI_4_], (I), and the mixed chloride/nitrate salt, bis­[2-methyl-3-(pyridin-2-yl)imidazo[1,5-*a*]pyridin-2-ium] 1.5-chlor­ide 0.5-nitrate trihydrate, 2C_13_H_12_N_3_^+^·1.5Cl^−^·0.5NO_3_^−^·3H_2_O, (II). Hybrid salt (I) crystallizes in the space group *P*2_1_/*n* with two [*L*]_2_[CdI_4_] mol­ecules in the asymmetric unit related by pseudosymmetry. In the crystal of (I), layers of organic cations and of tetra­halometallate anions are stacked parallel to the *ab* plane. Anti­parallel *L* ^+^ cations disposed in a herring-bone pattern form π-bonded chains through aromatic stacking. In the inorganic layer, adjacent tetra­hedral CdI_4_ units have no connectivity but demonstrate close packing of iodide anions. In the crystal lattice of (II), the cations are arranged in stacks propagating along the *a* axis; the one-dimensional hydrogen-bonded polymer built of chloride ions and water mol­ecules runs parallel to a column of stacked cations.

## Abstract 2

An organic–inorganic hybrid salt with two [*L*]_2_[CdI_4_] mol­ecules in the asymmetric unit related by pseudosymmetry exhibits a layered structure. In the mixed chloride/nitrate salt, the one-dimensional hydrogen-bonding polymer built of anions and water mol­ecules runs parallel to a column of stacked *L* ^+^ cations.

## Chemical context   

Organic–inorganic hybrid salts have maintained steady research inter­est in materials science (Díaz & Corma, 2018 ▸). By combining cation and anion networks in one continuous lattice, useful properties of organic and inorganic components are expected to translate into new multifunctional materials. Monovalent organic cations can form hybrid halometallates with halide anions and divalent metal ions with organic–inorganic Pb and Sn perovskites being the most investigated family because of their efficiency in solar cells (Brenner *et al.*, 2016 ▸). The exploration of hybrid compounds based on other polyhedra and connectivity through control of their chemical composition and structural dimensionality may bring applications in new areas of science and technology. Hybrid tetra­halometallates are a promising variety that can demonstrate properties of multiferroics (*β*-K_2_SeO_4_ analogues) and ionic liquids, show luminescence and a series of solid-phase transitions (García-Saiz *et al.*, 2014 ▸; Piecha-Bisiorek *et al.*, 2016 ▸; Jiang *et al.*, 2017 ▸).

The serendipitous discovery of the formation of 2-methyl-3-(pyridin-2-yl)imidazo[1,5-*a*]pyridinium cation, *L* ^+^, in the oxidative condensation–cyclization of 2-pyridine­carbaldehyde (2-PCA) and CH_3_NH_2_·HCl in methanol and the following preparation of the fluorescent [*L*]_2_[ZnCl_4_] hybrid salt in the presence of Zn^2+^ ions prompted our research on organic–inorganic halometalates with substituted imidazo[1,5-*a*]pyridinium cations (Buvaylo *et al.*, 2015 ▸; Vassilyeva *et al.*, 2019 ▸). The use of methyl­amine hydro­chloride instead of its conventional aqueous solution appeared to promote the cyclo­condensation with the formation of *L* ^+^ instead of the expected neutral Schiff base. Heterocycles with the imidazo[1,5-*a*]pyridine skeleton show prominent photophysical properties (Hutt *et al.*, 2012 ▸) and have the potential to be used in optoelectronic technology. Their incorporation in the halometallate structure may improve the mechanical properties, chemical resistance, thermal stability, *etc*. of organic materials.
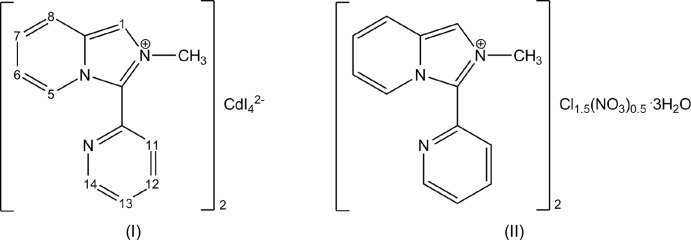


In the present work, we aimed to study the effect of the halide variation on the resulting hybrid salt structure. The new organic–inorganic hybrid [*L*]_2_[CdI_4_] (I) involving the *in situ-*formed *L* ^+^ cation has been prepared in the reaction system:

2-PCA – CH_3_NH_2_·HCl – CdI_2_ – KI – CH_3_OH

The use of Pb(NO_3_)_2_ in an attempt to synthesize a hybrid salt with an *L* ^+^ cation was not successful but led to the isolation of 2-methyl-3-(pyridin-2-yl)imidazo[1,5-*a*]pyridinium as a mixed chloride/nitrate salt, [*L*]_2_[Cl]_1.5_[NO_3_]_0.5_·3H_2_O (II) in the system:

2-PCA – CH_3_NH_2_·HCl – Pb(NO_3_)_2_ – CH_3_OH

The identities of the title compounds were confirmed by elemental analysis, IR and NMR spectroscopy, and single-crystal diffraction studies.

## Structural commentary   

**Figure 1:**
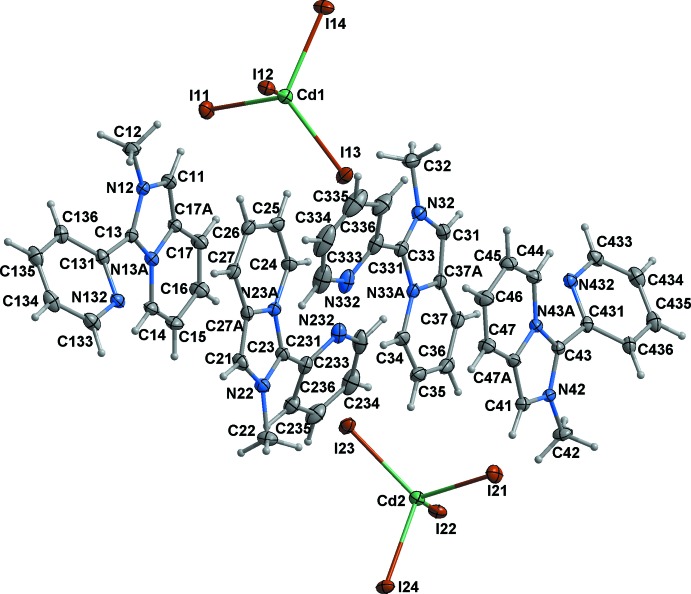
Mol­ecular structure and labelling of (I) with ellipsoids at the 50% probability level.

The hybrid salt (I) is built of discrete *L* ^+^ cations and CdI_4_^2–^ anions (Fig. 1 ▸). There are two symmetry-independent sets of (2*L* ^+^ + CdI_4_^2−^) ions related by pseudosymmetry in the asymmetric unit; *L* ^+^ cations in every set are crystallographically non-equivalent. They possess very similar structural configurations that are strictly comparable to those of the *L* ^+^ cations in ortho­rhom­bic [*L*]_2_[ZnCl_4_] and monoclinic [*L*]_2_[CoCl_4_] reported by us previously (Buvaylo *et al.*, 2015 ▸; Vassilyeva *et al.*, 2019 ▸). The replacement of chloride with iodide anions did not influence the stoichiometry of the resulting tetra­halometallate and the overall structure of the hybrid salt remained roughly the same.

The bond lengths of the pyridinium entities in the imidazo[1,5-*a*]pyridinium cores are as expected for such rings, the bond distances in the imidazolium rings fall in the range 1.350 (3)–1.409 (4) Å. The N12 and N13*A*, N22 and N23*A*, N32 and N33*A*, N42 and N43*A* atoms are planar with the sum of three angles being 360°. The fused cores of all four *L* ^+^ cations are virtually coplanar: the dihedral angles between the five- and six-membered rings vary from 1.22 to 2.26°. The pendant pyridyl rings are twisted by approximately 25.60–38.52° with respect to the imidazo[1,5-*a*]pyridinium cores. The 2-methyl-3-(pyridin-2-yl)imidazo[1,5-*a*]pyridinium units are mono-cationic and aromatic with the positive charge being delocalized on atoms N12 and N13*A*, N22 and N23*A*, N32 and N33*A*, N42 and N43*A*.

The tetra­hedral CdI_4_^2–^ anions are moderately distorted: the Cd—I distances lie in the range 2.7573 (3)–2.8023 (3) Å while the I—Cd—I angles vary from 102.186 (8) to 117.300 (9)° (Table 1 ▸). The average Cd—I distance of 2.78 Å is comparable to those found in the CSD (version 5.40 of November 2018; Groom *et al.*, 2016 ▸) for other Cd^II^ salts containing isolated CdI_4_^2–^ tetra­hedral anions (an average of 2.777 (3) Å for Cd—I with a range of 2.684–2.827 Å).

**Figure 2:**
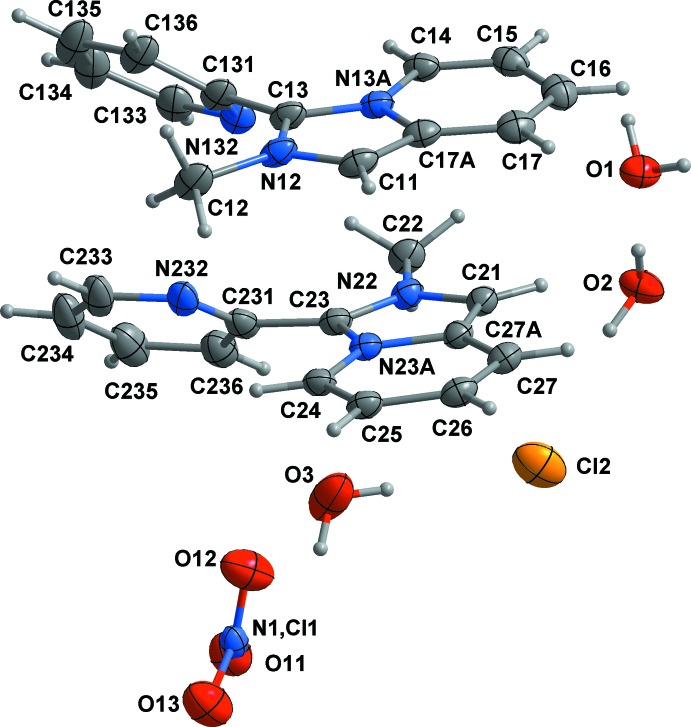
Mol­ecular structure and labelling of (II) with ellipsoids at the 50% probability level.

[*L*]_2_[Cl]_1.5_[NO_3_]_0.5_·3H_2_O (II) crystallizes in the triclinic space group and is isomorphous with [*L*][Cl]·1.5H_2_O (CSD refcode HUMCUP; Buvaylo *et al.*, 2015 ▸). There are two crystallographically non-equivalent *L* ^+^ cations, *L*1 (N12, N13*A*) and *L*2 (N22, N23*A*), 1.5 chloride and 0.5 nitrate anions, and three water mol­ecules of crystallization in the asymmetric unit (Fig. 2 ▸). The bond lengths and angles of two independent *L* ^+^ cations with planar fused cores (dihedral angles for *L*1 and *L*2 are about 0.88 and 1.45°, respectively) are very similar to those in (I). The twist of the pendant pyridyl rings with respect to the planes of the remainder of the cations is, however, more pronounced in (II): approximately 43.21 and 40.92° for *L*1 and *L*2, respectively.

## Supra­molecular features   

**Figure 3:**
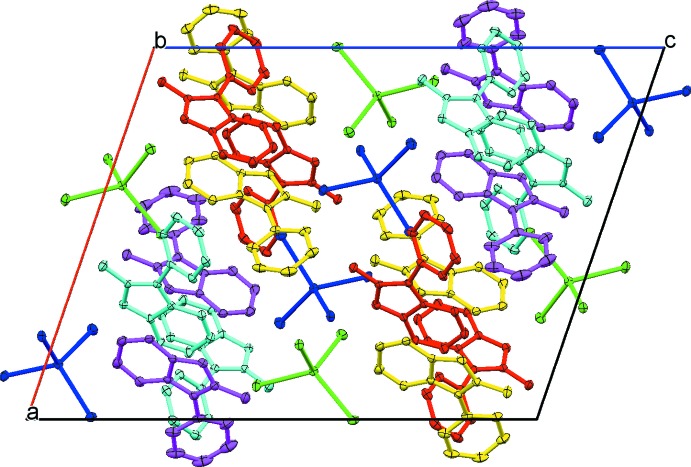
Crystal packing of (I) viewed along the *b* axis, showing the alternation of cation and anion layers. Symmetry-independent *L* ^+^ cations and CdI_4_^2–^ anions are drawn with different colours; H atoms are not shown.

**Figure 4:**
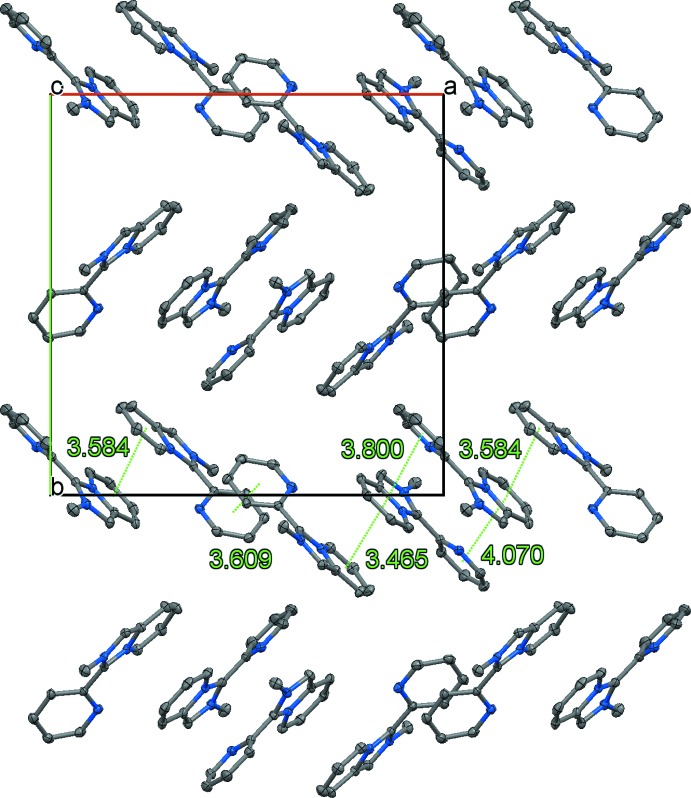
Organic layer in (I) viewed along the *c* axis, showing π-bonded chains of anti­parallel *L* ^+^ cations disposed in a herringbone pattern.

Compound (I) exhibits a pseudo-layered structure with layers of organic cations and of tetra­iodo­cadmate anions stacked parallel to the *ab* plane (Fig. 3 ▸). In a layer, *L* ^+^ cations disposed in an anti­parallel fashion adopt a herring-bone pattern and form π-bonded chains through three types of stacking contacts (Fig. 4 ▸). Those involve the six-membered rings of neighbouring mol­ecules, pendant pyridyl rings, and π–π inter­actions between the former and the latter. The π-stacking is offset by about half a ring diameter with centroid–centroid distances in the range 3.465 (2)–4.070 (2) Å.

In the inorganic layer, the adjacent CdI_4_ units have no connectivity with the minimum Cd⋯Cd distance being 8.943 Å. The halide anions, however, demonstrate close packing: the shortest distance between I atoms on adjacent anions of 4.192 Å is smaller than double the iodide Shannon (1976 ▸) ionic radius [2 × *r*(I^−^) = 4.40 Å]. The separation between two consecutive inorganic planes corresponds to half the cell length of the *c* axis (11.220 Å).

Classical hydrogen-bonding inter­actions are absent in (I). Numerous C—H⋯I—Cd contacts between the organic and inorganic counterparts with H⋯I distances in the range 2.93–3.22 Å are too weak and mostly result from van der Waals close packing. Such a structural feature is commonly observed in organic–inorganic hybrid iodo­metallates (Chen *et al.*, 2010 ▸; Li *et al.*, 2018 ▸).

**Figure 5:**
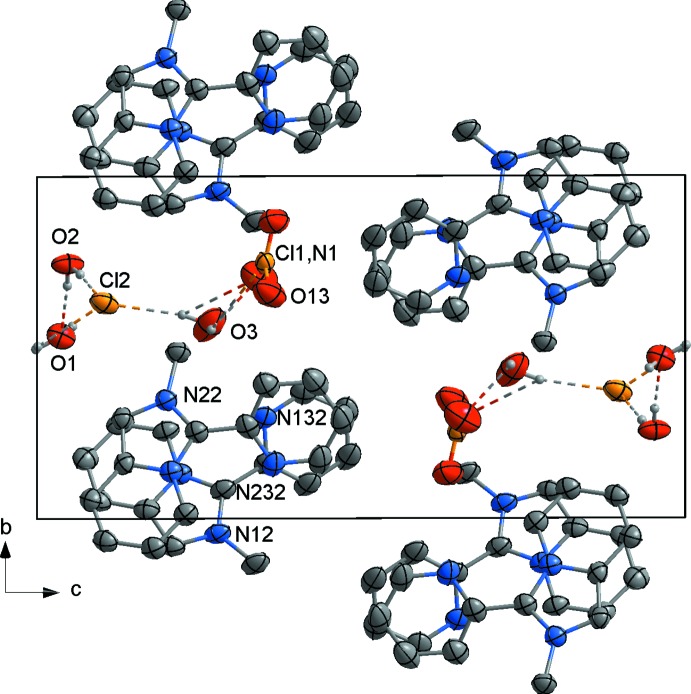
The unit-cell contents of (II) projected along the *a* axis, showing the stacking of *L* ^+^ cations and the formation of a hydrogen-bonded polymer *via* O—H⋯Cl and O—H⋯O inter­actions. The C-bound H atoms are not shown.

In the crystal lattice of (II), the alternating *L*1 and *L*2 cations are arranged in stacks aligned along the *a-*axis direction (Fig. 5 ▸) with almost coplanar fused cores of adjacent mol­ecules (dihedral angle about 4.87°). The pendant pyridyl rings on neighbouring cations are twisted by approximately 16° with respect to each other and display aromatic stacking with ring-centroid distances of 3.675 (2) and 3.798 (2) Å. The chloride ions and water mol­ecules are involved in hydrogen bonding, forming a one-dimensional hydrogen-bonded polymer that runs parallel to a column of stacked cations (Fig. 5 ▸, Table 2 ▸).

## Database survey   

Apart from [*L*][Cl]·1.5H_2_O and four chloro­metallates [*L*]_2_[*M*Cl_4_], [*ML*Cl_3_] (*M* = Co^II^ and Zn^II^) published by our research group, there are no compounds containing the *L* ^+^ cation in the CSD (version 5.40 of November 2018; Groom *et al.*, 2016 ▸). The structures in which the imidazo[1,5-*a*]pyridinium core is comparable with the title compounds are limited to a handful of organic salts with varying substituents in the imidazolium ring. The most similar to (II) are 2-[2-(1*H*-imidazol-3-ium-5-yl)eth­yl]-3-(pyridin-2-yl)imidazo[1,5-*a*]pyridin-2-ium diperchlorate (CSD refcode UREYIA; Türkyilmaz *et al.*, 2011 ▸) and 2-(2-pyrid­yl)-*N* ^3^-(4-chloro­phen­yl)imidazo[1,5-*a*]pyridinium perchlorate (YIHFEB; Mitra *et al.*, 2007 ▸) having ethyl­imidazolium and chloro­phenyl substit­uents, respectively, instead of the methyl group in *L* ^+^. The neutral mol­ecule of *L* lacking the methyl group was also reported (PRIMPY; Shibahara *et al.*, 2006 ▸). It crystallizes in the ortho­rhom­bic space group *P*2_1_2_1_2_1_ and is able to act as a κ^2^(*N*,*N*) chelate ligand forming an Mn^II^ complex (Álvarez *et al.*, 2012 ▸). Inter­estingly, 3-(pyridin-2-yl)imidazo[1,5-*a*]pyridine could be easily separated from the metal by boiling the complex suspension in water.

## Synthesis and crystallization   

***Synthesis of [L]_2_[CdI_4_] (I)*** **:** 2-PCA (0.38 ml, 4 mmol) was stirred with CH_3_NH_2_·HCl (0.27 g, 4 mmol) in 20 ml of methanol in a 50 ml conical flask at room temperature (r.t.) for half an hour. The resultant yellow solution was left in the open air overnight and turned olive. Dry CdI_2_ (0.37 g, 1 mmol) and KI (0.33 g, 2 mmol) were added to the ligand solution and the mixture was heated slightly and stirred magnetically for half an hour to ensure salt dissolution. The resulting brownish solution was filtered and left to evaporate at r.t. Pale-brown prisms of (I) suitable for X-ray crystallography formed within two days. The crystals were filtered off, washed with diethyl ether and finally dried in air. More product was obtained upon slow evaporation in air of the mother liquor. Yield: 65% (based on cadmium). Analysis calculated for C_26_H_24_I_4_N_6_Cd (1040.51): C, 30.01; H 2.32; N 8.08%. Found: C 30.36; H 2.04; N 8.24%. FT–IR (ν, cm^−1^): 3436br, 3138, 3116, 3056, 2994, 2924, 1652, 1582, 1518, 1464, 1446, 1424, 1366, 1334, 1286, 1250, 1180, 1154, 1104, 1054, 1038, 990, 942, 778, 742, 658, 610, 568, 556, 430, 404. ^1^H NMR (400 MHz, DMSO-*d*_6_): δ (ppm) 8.92 (*d*, 1H, *J* = 4.4 Hz, H14), 8.70 (*d*, 1H, *J* = 7.3 Hz, H5), 8.60 (*s*, 1H, H1), 8.25–8.17 (*m*, 2H, H11+H12) , 8.02 (*d*, 1H, *J* = 9.3 Hz, H8), 7.76–7.73 (*m*, 1H, H13), 7.37 (*t*, 1H, *J* = 8.1 Hz, H7), 7.23 (*t*, 1H, *J* = 6.6 Hz, H6), 4.30 (*s*, 3H, CH_3_).

***Synthesis of [L]_2_[Cl]_1.5_[NO_3_]_0.5_·3H_2_O (II)*** **:** 2-PCA (0.38 ml, 4 mmol) was stirred with CH_3_NH_2_·HCl (0.27 g, 4 mmol) in 20 ml methanol in a 50 ml conical flask at r.t. for half an hour. Dry Pb(NO_3_)_2_ (0.33 g, 1 mmol) was added to this solution and the mixture was stirred magnetically for another hour under mild heating to ensure salt dissolution. The yellow solution that became turbid was filtered and left to evaporate. Light-brown needles of (II) formed next day. They were filtered off, washed with diethyl ether and dried in air. Yield 51% (based on 2-PCA). Analysis calculated for C_26_H_30_Cl_1.5_N_6.5_O_4.5_ (558.74): C 55.89; H 5.41; N 16.29%. Found: C 54.75; H 5.66; N 15.67%. FT–IR (ν, cm^−1^): 3450br, 3142, 3094, 3062, 3040, 1652, 1604, 1586, 1520, 1470, 1388(NO_3_), 1364, 1334, 1302, 1250, 1180, 1160, 1100, 1054, 1040, 992, 944, 800, 780, 748, 666, 622, 610, 568, 558, 434, 408. ^1^H NMR (400 MHz, DMSO-*d*_6_/CCl_4_): δ (ppm) 8.94 (*d*, 1H, *J* = 4.9 Hz, H14), 8.72 (*d*, 1H, *J* = 6.8 Hz, H5), 8.59 (*s*, 1H, H1), 8.25–8.18 (*m*, 2H, H11+H12), 8.03 (*d*, 1H, *J* = 9.3 Hz, H8), 7.76 (*t*, 1H, *J* = 5.6 Hz, H13), 7.39 (*t*, 1H, *J* = 7.8 Hz, H7), 7.25 (*t*, 1H, *J* = 6.8 Hz, H6), 4.31 (*s*, 3H, CH_3_).

The compounds are soluble in water, alcohols, dmf and dmso. The hybrid salt (I) is stable in air for months, while (II) appears moisture sensitive. Medium intensity peaks above 3000 cm^−1^ and medium or strong peaks in the range 1650–1450 cm^−1^ in the IR spectra of (I) and (II) indicate the presence of aromatic rings. The presence of alkyl groups is confirmed by the medium-strength bands in the range 3000–2800 cm^−1^. A very strong band at 1388 cm^−1^ in the spectrum of (II) originates from vibration of the NO_3_^−^ ion. The ^1^H NMR spectra in DMSO-*d*_6_ at room temperature showed the correct pyrid­yl/alkyl proton ratios of *L* ^+^ cation for (I) and (II).

## Refinement   

Crystal data, data collection and structure refinement details for both structures are summarized in Table 3 ▸. Compound (I) crystallizes with two [*L*]_2_[CdI_4_] mol­ecules in the asymmetric unit. The *checkCIF* implementation of *PLATON* ADDSYM detects an additional (pseudo) symmetry element, *c*/2, with a 91% fit and suggests that the length of the *c* axis should be halved. This is pseudosymmetry as seen in projections down the *a* and *b* axes and also by noting that the number of reflections with significant intensity being much greater than half the total number (23740 out of 31421). For (II), the cell setting used is that of the isomorphous chloride HUMCUP. One anion site in (II) was modelled as being disordered between a Cl^−^ and a NO_3_^−^ ion with site occupancies constrained to 0.5 after trial refinement. The water mol­ecule hydrogen atoms in (II) were located and refined with geometries restrained to ideal values. All remaining hydrogen atoms in (I) and (II) were added at calculated positions and refined by use of a riding model with isotropic displacement parameters based on those of the parent atom (C—H = 0.95 Å, *U*_iso_(H) = 1.2*U*_eq_C for CH, C—H = 0.98 Å, *U*_iso_(H) = 1.5*U*_eq_C for CH_3_).

## Supplementary Material

Crystal structure: contains datablock(s) I, II. DOI: 10.1107/S2056989019009964/lh5912sup1.cif

Structure factors: contains datablock(s) I. DOI: 10.1107/S2056989019009964/lh5912Isup2.hkl

Structure factors: contains datablock(s) II. DOI: 10.1107/S2056989019009964/lh5912IIsup3.hkl

IR spectrum of (I). DOI: 10.1107/S2056989019009964/lh5912sup4.pdf

IR spectrum of (II). DOI: 10.1107/S2056989019009964/lh5912sup5.pdf

CCDC references: 1940074, 1940074, 1940075

Additional supporting information:  crystallographic information; 3D view; checkCIF report

## Figures and Tables

**Table 1:** Selected geometric parameters (Å, °) for (I)

Cd1—I14	2.7573 (3)	Cd2—I24	2.7575 (3)
Cd1—I11	2.7764 (3)	Cd2—I21	2.7610 (3)
Cd1—I13	2.7949 (3)	Cd2—I22	2.7943 (3)
Cd1—I12	2.8023 (3)	Cd2—I23	2.7958 (3)
			
I14—Cd1—I11	107.058 (9)	I24—Cd2—I21	105.915 (9)
I14—Cd1—I13	115.503 (10)	I24—Cd2—I22	105.260 (8)
I11—Cd1—I13	103.726 (9)	I21—Cd2—I22	116.026 (10)
I14—Cd1—I12	102.186 (8)	I24—Cd2—I23	112.854 (9)
I11—Cd1—I12	117.300 (9)	I21—Cd2—I23	108.090 (9)
I13—Cd1—I12	111.499 (9)	I22—Cd2—I23	108.791 (9)

**Table 2:** Hydrogen-bond geometry (Å, °) for (II)

*D*—H⋯*A*	*D*—H	H⋯*A*	*D*⋯*A*	*D*—H⋯*A*
O1—H1*AO*⋯Cl2^i^	0.836 (18)	2.339 (18)	3.174 (2)	176 (3)
O1—H1*BO*⋯Cl2^ii^	0.832 (18)	2.41 (2)	3.229 (2)	171 (4)
O2—H2*AO*⋯O1	0.832 (17)	1.925 (18)	2.755 (3)	175 (4)
O2—H2*BO*⋯Cl2	0.832 (18)	2.352 (18)	3.178 (2)	172 (4)
O3—H3*AO*⋯Cl1	0.802 (19)	2.95 (5)	3.398 (4)	118 (4)
O3—H3*BO*⋯Cl2	0.848 (18)	2.33 (2)	3.166 (3)	168 (5)
O3—H3*AO*⋯O12	0.802 (19)	2.10 (5)	2.363 (7)	99 (4)
C11—H11⋯Cl2^iii^	0.95	2.71	3.640 (3)	166
C12—H12*A*⋯Cl1^iv^	0.98	2.79	3.638 (4)	146
C14—H14⋯N132	0.95	2.53	3.024 (4)	112
C14—H14⋯O3^i^	0.95	2.47	3.330 (4)	151
C15—H15⋯Cl2^i^	0.95	2.75	3.671 (3)	165
C17—H17⋯O2^iii^	0.95	2.57	3.244 (3)	128
C24—H24⋯N232	0.95	2.51	3.019 (4)	114
C27—H27⋯O2^ii^	0.95	2.48	3.255 (3)	139
C236—H236⋯O3	0.95	2.35	3.160 (5)	143

Symmetry codes: (i) 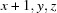; (ii) 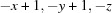; (iii) 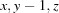; (iv) 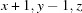.

**Table 3:** Experimental details

	(I)	(II)
Crystal data		
Chemical formula	(C_13_H_12_N_3_)_2_[CdI_4_]	2C_13_H_12_N_3_^+^·1.5Cl^−^·0.5NO_3_^−^·3H_2_O
*M*_r_	1040.51	558.74
Crystal system, space group	Monoclinic, *P*2_1_/*n*	Triclinic, *P* 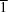
Temperature (K)	100	100
*a*, *b*, *c* (Å)	17.2718 (2), 16.6530 (1), 22.4402 (2)	7.3959 (5), 10.2889 (8), 18.5155 (10)
α, β, γ (°)	90, 108.922 (1), 90	88.208 (5), 95.033 (5), 108.916 (5)
*V* (Å^3^)	6105.62 (10)	1327.71 (16)
*Z*	8	2
Radiation type	Mo *K*α	Cu *K*α
μ (mm^−1^)	4.79	2.14
Crystal size (mm)	0.45 × 0.27 × 0.25	0.23 × 0.05 × 0.03

Data collection		
Diffractometer	Oxford Diffraction Gemini	Oxford Diffraction Gemini
Absorption correction	Analytical (*CrysAlis PRO*; Rigaku OD, 2016 ▸)	Analytical (*CrysAlis PRO*; Rigaku OD, 2016 ▸)
*T*_min_, *T*_max_	0.248, 0.433	0.777, 0.942
No. of measured, independent and observed [*I* > 2σ(*I*)] reflections	207318, 31421, 23740	11325, 4693, 3366
*R*_int_	0.048	0.050
(sin θ/λ)_max_ (Å^−1^)	0.859	0.598

Refinement		
*R*[*F* ^2^ > 2σ(*F* ^2^)], *wR*(*F* ^2^), *S*	0.035, 0.096, 1.01	0.047, 0.121, 1.02
No. of reflections	31421	4693
No. of parameters	671	385
No. of restraints	0	9
H-atom treatment	H-atom parameters constrained	H atoms treated by a mixture of independent and constrained refinement
Δρ_max_, Δρ_min_ (e Å^−3^)	2.66, −2.53	0.26, −0.26

Computer programs: *CrysAlis PRO* (Rigaku OD, 2016 ▸), *SIR92* (Altomare *et al.*, 1994 ▸), *SHELXT* (Sheldrick, 2015*a* ▸), *SHELXL2014* (Sheldrick, 2015*b* ▸), *DIAMOND* (Brandenburg, 1999 ▸), *Mercury* (Macrae *et al.*, 2006 ▸) and *WinGX* (Farrugia, 2012 ▸).

## supplementary crystallographic information

### Bis[2-methyl-3-(pyridin-2-yl)imidazo[1,5-a]pyridin-2-ium] tetraiodocadmate (I) Crystal data

**Table:**

(C_13_H_12_N_3_)_2_[CdI_4_]	*F*(000) = 3856
*M**_r_* = 1040.51	*D*_x_ = 2.264 Mg m^−^^3^
Monoclinic, *P*2_1_/*n*	Mo *K*α radiation, λ = 0.71073 Å
*a* = 17.2718 (2) Å	Cell parameters from 65440 reflections
*b* = 16.6530 (1) Å	θ = 2.3–37.2°
*c* = 22.4402 (2) Å	µ = 4.79 mm^−^^1^
β = 108.922 (1)°	*T* = 100 K
*V* = 6105.62 (10) Å^3^	Prism, pale brown
*Z* = 8	0.45 × 0.27 × 0.25 mm

### Bis[2-methyl-3-(pyridin-2-yl)imidazo[1,5-a]pyridin-2-ium] tetraiodocadmate (I) Data collection

**Table:**

Oxford Diffraction Gemini diffractometer	31421 independent reflections
Radiation source: normal-focus sealed tube	23740 reflections with *I* > 2σ(*I*)
Graphite monochromator	*R*_int_ = 0.048
Detector resolution: 10.4738 pixels mm^-1^	θ_max_ = 37.7°, θ_min_ = 2.3°
ω scans	*h* = −29→29
Absorption correction: analytical (CrysAlis Pro; Rigaku OD, 2016)	*k* = −27→28
*T*_min_ = 0.248, *T*_max_ = 0.433	*l* = −38→38
207318 measured reflections	

### Bis[2-methyl-3-(pyridin-2-yl)imidazo[1,5-a]pyridin-2-ium] tetraiodocadmate (I) Refinement

**Table:**

Refinement on *F*^2^	0 restraints
Least-squares matrix: full	Hydrogen site location: inferred from neighbouring sites
*R*[*F*^2^ > 2σ(*F*^2^)] = 0.035	H-atom parameters constrained
*wR*(*F*^2^) = 0.096	*w* = 1/[σ^2^(*F*_o_^2^) + (0.045*P*)^2^ + 12.*P*] where *P* = (*F*_o_^2^ + 2*F*_c_^2^)/3
*S* = 1.01	(Δ/σ)_max_ = 0.003
31421 reflections	Δρ_max_ = 2.66 e Å^−^^3^
671 parameters	Δρ_min_ = −2.53 e Å^−^^3^

### Bis[2-methyl-3-(pyridin-2-yl)imidazo[1,5-a]pyridin-2-ium] tetraiodocadmate (I) Special details

**Table:**

Geometry. All esds (except the esd in the dihedral angle between two l.s. planes) are estimated using the full covariance matrix. The cell esds are taken into account individually in the estimation of esds in distances, angles and torsion angles; correlations between esds in cell parameters are only used when they are defined by crystal symmetry. An approximate (isotropic) treatment of cell esds is used for estimating esds involving l.s. planes.
Refinement. The largest peak is 0.57 Angstroms from I13; the deepest hole is 0.39 Angstroms from I14.

### Bis[2-methyl-3-(pyridin-2-yl)imidazo[1,5-a]pyridin-2-ium] tetraiodocadmate (I) Fractional atomic coordinates and isotropic or equivalent isotropic displacement parameters (Å^2^)

**Table:**

	*x*	*y*	*z*	*U*_iso_*/*U*_eq_	
Cd1	0.37091 (2)	0.23322 (2)	0.02990 (2)	0.01602 (4)	
I11	0.27236 (2)	0.11998 (2)	0.06096 (2)	0.02197 (4)	
I12	0.29968 (2)	0.38160 (2)	−0.01581 (2)	0.01727 (3)	
I13	0.50281 (2)	0.25114 (2)	0.14100 (2)	0.02218 (4)	
I14	0.40935 (2)	0.17338 (2)	−0.07161 (2)	0.02338 (4)	
Cd2	0.64769 (2)	0.76930 (2)	0.47496 (2)	0.01661 (4)	
I21	0.74129 (2)	0.87369 (2)	0.43129 (2)	0.02373 (4)	
I22	0.72313 (2)	0.62525 (2)	0.52637 (2)	0.01842 (4)	
I23	0.50554 (2)	0.73423 (2)	0.37519 (2)	0.02152 (4)	
I24	0.61626 (2)	0.84767 (2)	0.57291 (2)	0.02040 (4)	
C11	0.19207 (18)	0.32624 (17)	0.10784 (13)	0.0184 (5)	
H11	0.2047	0.2987	0.0751	0.022*	
N12	0.13723 (15)	0.38690 (14)	0.10047 (11)	0.0158 (4)	
C12	0.0920 (2)	0.4207 (2)	0.03863 (14)	0.0230 (6)	
H12A	0.0809	0.4777	0.0433	0.034*	
H12B	0.0401	0.3919	0.0208	0.034*	
H12C	0.1247	0.4152	0.0104	0.034*	
C13	0.13355 (17)	0.41169 (16)	0.15683 (13)	0.0156 (4)	
N13A	0.18897 (15)	0.36683 (14)	0.20083 (11)	0.0164 (4)	
C14	0.2109 (2)	0.36855 (18)	0.26667 (13)	0.0199 (5)	
H14	0.1853	0.4051	0.2869	0.024*	
C15	0.2689 (2)	0.31740 (19)	0.30068 (14)	0.0220 (5)	
H15	0.2838	0.3180	0.3453	0.026*	
C16	0.3086 (2)	0.2622 (2)	0.27127 (15)	0.0245 (6)	
H16	0.3498	0.2273	0.2964	0.029*	
C17	0.2875 (2)	0.25948 (19)	0.20740 (14)	0.0209 (5)	
H17	0.3138	0.2230	0.1876	0.025*	
C17A	0.22563 (18)	0.31209 (16)	0.17076 (13)	0.0160 (4)	
C131	0.07949 (18)	0.47317 (17)	0.16853 (14)	0.0179 (5)	
N132	0.11393 (17)	0.51982 (15)	0.21937 (12)	0.0196 (4)	
C133	0.0665 (2)	0.57605 (19)	0.23213 (16)	0.0242 (6)	
H133	0.0893	0.6085	0.2684	0.029*	
C134	−0.0138 (2)	0.58999 (19)	0.19568 (17)	0.0256 (6)	
H134	−0.0443	0.6324	0.2057	0.031*	
C135	−0.0492 (2)	0.54062 (19)	0.14400 (16)	0.0249 (6)	
H135	−0.1045	0.5481	0.1184	0.030*	
C136	−0.00187 (18)	0.47978 (18)	0.13040 (15)	0.0195 (5)	
H136	−0.0245	0.4440	0.0962	0.023*	
C21	0.34085 (19)	0.55077 (18)	0.31285 (14)	0.0202 (5)	
H21	0.3088	0.5934	0.3207	0.024*	
N22	0.39912 (16)	0.50795 (15)	0.35691 (11)	0.0183 (4)	
C22	0.4268 (2)	0.5301 (2)	0.42410 (14)	0.0245 (6)	
H22A	0.4855	0.5193	0.4425	0.037*	
H22B	0.3967	0.4984	0.4462	0.037*	
H22C	0.4165	0.5874	0.4283	0.037*	
C23	0.43209 (17)	0.45102 (16)	0.32954 (13)	0.0167 (5)	
N23A	0.39559 (15)	0.45990 (14)	0.26613 (11)	0.0166 (4)	
C24	0.40995 (19)	0.41887 (18)	0.21672 (14)	0.0201 (5)	
H24	0.4498	0.3774	0.2246	0.024*	
C25	0.3656 (2)	0.4397 (2)	0.15704 (14)	0.0236 (6)	
H25	0.3755	0.4127	0.1229	0.028*	
C26	0.3049 (2)	0.5005 (2)	0.14408 (15)	0.0235 (6)	
H26	0.2746	0.5134	0.1017	0.028*	
C27	0.29006 (19)	0.54049 (19)	0.19192 (14)	0.0207 (5)	
H27	0.2491	0.5809	0.1836	0.025*	
C27A	0.33702 (17)	0.52055 (18)	0.25455 (14)	0.0182 (5)	
C231	0.49081 (18)	0.38885 (17)	0.36104 (14)	0.0190 (5)	
N232	0.53859 (17)	0.36191 (16)	0.32836 (14)	0.0231 (5)	
C233	0.5925 (2)	0.3044 (2)	0.35546 (19)	0.0286 (7)	
H233	0.6259	0.2838	0.3327	0.034*	
C234	0.6028 (2)	0.2729 (2)	0.4147 (2)	0.0324 (8)	
H234	0.6437	0.2337	0.4326	0.039*	
C235	0.5522 (2)	0.2998 (2)	0.44706 (17)	0.0302 (7)	
H235	0.5571	0.2789	0.4875	0.036*	
C236	0.4941 (2)	0.35806 (19)	0.41939 (15)	0.0232 (6)	
H236	0.4572	0.3765	0.4400	0.028*	
C31	0.65090 (19)	0.43165 (19)	0.19254 (14)	0.0203 (5)	
H31	0.6798	0.3900	0.1799	0.024*	
N32	0.59055 (17)	0.47812 (16)	0.15388 (12)	0.0204 (5)	
C32	0.5572 (2)	0.4634 (2)	0.08537 (14)	0.0274 (6)	
H32A	0.5649	0.4068	0.0767	0.041*	
H32B	0.5858	0.4974	0.0636	0.041*	
H32C	0.4986	0.4763	0.0703	0.041*	
C34	0.6009 (2)	0.55503 (19)	0.30350 (15)	0.0232 (6)	
H34	0.5628	0.5971	0.3008	0.028*	
N33A	0.60665 (15)	0.51811 (15)	0.24932 (12)	0.0176 (4)	
C33	0.56372 (18)	0.53278 (17)	0.18755 (14)	0.0187 (5)	
C35	0.6512 (2)	0.5294 (2)	0.36002 (15)	0.0269 (6)	
H35	0.6474	0.5536	0.3973	0.032*	
C36	0.7097 (2)	0.4673 (2)	0.36529 (15)	0.0268 (6)	
H36	0.7447	0.4511	0.4056	0.032*	
C37	0.71557 (19)	0.4317 (2)	0.31319 (14)	0.0228 (6)	
H37	0.7548	0.3906	0.3163	0.027*	
C37A	0.66222 (18)	0.45634 (18)	0.25344 (14)	0.0185 (5)	
C331	0.50283 (19)	0.59623 (18)	0.16398 (16)	0.0238 (6)	
N332	0.46510 (18)	0.62050 (16)	0.20472 (16)	0.0284 (6)	
C333	0.4092 (2)	0.6785 (2)	0.1857 (2)	0.0381 (9)	
H333	0.3830	0.6968	0.2144	0.046*	
C334	0.3872 (3)	0.7132 (2)	0.1272 (3)	0.0465 (12)	
H334	0.3454	0.7528	0.1152	0.056*	
C335	0.4270 (3)	0.6896 (2)	0.0863 (2)	0.0445 (11)	
H335	0.4136	0.7131	0.0456	0.053*	
C336	0.4875 (3)	0.6304 (2)	0.10500 (19)	0.0347 (8)	
H336	0.5173	0.6142	0.0781	0.042*	
C41	0.80926 (18)	0.65616 (16)	0.38757 (13)	0.0167 (4)	
H41	0.7995	0.6836	0.4216	0.020*	
N42	0.86433 (15)	0.59627 (14)	0.39249 (10)	0.0154 (4)	
C42	0.9130 (2)	0.5618 (2)	0.45322 (14)	0.0236 (6)	
H42A	0.9235	0.5049	0.4476	0.035*	
H42B	0.8829	0.5669	0.4833	0.035*	
H42C	0.9652	0.5905	0.4693	0.035*	
C43	0.86380 (17)	0.57178 (16)	0.33500 (12)	0.0153 (4)	
N43A	0.80540 (15)	0.61581 (14)	0.29262 (11)	0.0154 (4)	
C44	0.77946 (19)	0.61436 (18)	0.22676 (13)	0.0190 (5)	
H44	0.8040	0.5788	0.2050	0.023*	
C45	0.7187 (2)	0.6647 (2)	0.19465 (14)	0.0226 (6)	
H45	0.7008	0.6644	0.1499	0.027*	
C46	0.6809 (2)	0.7183 (2)	0.22663 (15)	0.0244 (6)	
H46	0.6374	0.7520	0.2030	0.029*	
C47A	0.77035 (18)	0.66993 (17)	0.32480 (13)	0.0168 (5)	
C47	0.7067 (2)	0.72139 (19)	0.29039 (14)	0.0220 (5)	
H47	0.6823	0.7577	0.3116	0.026*	
C431	0.91603 (17)	0.51078 (16)	0.32062 (13)	0.0162 (4)	
N432	0.87936 (16)	0.46557 (15)	0.26967 (12)	0.0182 (4)	
C433	0.9247 (2)	0.40867 (18)	0.25464 (15)	0.0219 (5)	
H433	0.9001	0.3769	0.2182	0.026*	
C434	1.0058 (2)	0.39372 (19)	0.28947 (16)	0.0242 (6)	
H434	1.0350	0.3512	0.2783	0.029*	
C435	1.0435 (2)	0.4425 (2)	0.34129 (16)	0.0243 (6)	
H435	1.0991	0.4341	0.3658	0.029*	
C436	0.99816 (18)	0.50336 (18)	0.35640 (14)	0.0196 (5)	
H436	1.0226	0.5390	0.3903	0.024*	

### Bis[2-methyl-3-(pyridin-2-yl)imidazo[1,5-a]pyridin-2-ium] tetraiodocadmate (I) Atomic displacement parameters (Å^2^)

**Table:**

	*U*^11^	*U*^22^	*U*^33^	*U*^12^	*U*^13^	*U*^23^
Cd1	0.01811 (9)	0.01385 (8)	0.01690 (8)	0.00019 (6)	0.00679 (7)	0.00022 (6)
I11	0.02464 (9)	0.01729 (8)	0.02594 (9)	−0.00521 (6)	0.01090 (7)	0.00041 (6)
I12	0.02242 (9)	0.01406 (7)	0.01630 (7)	0.00214 (6)	0.00761 (6)	0.00116 (5)
I13	0.01875 (8)	0.01961 (8)	0.02435 (9)	0.00307 (6)	0.00169 (7)	−0.00509 (6)
I14	0.03063 (11)	0.02327 (9)	0.01615 (8)	0.00862 (7)	0.00748 (7)	0.00058 (6)
Cd2	0.01928 (9)	0.01392 (8)	0.01732 (8)	0.00019 (6)	0.00691 (7)	−0.00021 (6)
I21	0.02773 (10)	0.02007 (8)	0.02569 (9)	−0.00627 (7)	0.01185 (8)	0.00100 (7)
I22	0.02594 (9)	0.01397 (7)	0.01642 (7)	0.00303 (6)	0.00836 (6)	0.00036 (5)
I23	0.01884 (9)	0.01971 (8)	0.02393 (9)	0.00086 (6)	0.00406 (7)	−0.00565 (6)
I24	0.02381 (9)	0.01991 (8)	0.01579 (7)	0.00374 (6)	0.00408 (6)	−0.00304 (6)
C11	0.0186 (13)	0.0197 (12)	0.0178 (11)	0.0006 (9)	0.0072 (10)	−0.0014 (9)
N12	0.0141 (10)	0.0177 (10)	0.0150 (9)	−0.0021 (8)	0.0041 (8)	−0.0014 (7)
C12	0.0226 (14)	0.0301 (15)	0.0147 (11)	0.0048 (11)	0.0039 (10)	0.0010 (10)
C13	0.0142 (11)	0.0163 (10)	0.0168 (11)	−0.0019 (8)	0.0058 (9)	−0.0010 (8)
N13A	0.0165 (10)	0.0185 (10)	0.0157 (9)	−0.0005 (8)	0.0072 (8)	0.0001 (7)
C14	0.0232 (14)	0.0230 (13)	0.0158 (11)	−0.0031 (10)	0.0098 (10)	−0.0013 (9)
C15	0.0266 (15)	0.0259 (14)	0.0140 (11)	0.0009 (11)	0.0072 (10)	0.0021 (10)
C16	0.0276 (16)	0.0253 (14)	0.0201 (13)	0.0067 (12)	0.0071 (11)	0.0064 (10)
C17	0.0217 (14)	0.0236 (13)	0.0185 (12)	0.0059 (10)	0.0079 (10)	0.0042 (10)
C17A	0.0168 (12)	0.0175 (11)	0.0151 (10)	0.0012 (9)	0.0069 (9)	0.0005 (8)
C131	0.0182 (12)	0.0163 (11)	0.0212 (12)	−0.0008 (9)	0.0092 (10)	0.0007 (9)
N132	0.0220 (12)	0.0194 (10)	0.0207 (11)	−0.0032 (9)	0.0116 (9)	−0.0028 (8)
C133	0.0304 (16)	0.0190 (12)	0.0298 (15)	−0.0021 (11)	0.0190 (13)	−0.0019 (11)
C134	0.0296 (16)	0.0173 (12)	0.0365 (17)	0.0017 (11)	0.0201 (14)	0.0049 (11)
C135	0.0231 (15)	0.0233 (13)	0.0325 (16)	0.0067 (11)	0.0146 (13)	0.0077 (12)
C136	0.0160 (12)	0.0191 (12)	0.0249 (13)	0.0000 (9)	0.0086 (10)	0.0021 (10)
C21	0.0196 (13)	0.0215 (12)	0.0211 (12)	0.0063 (10)	0.0089 (10)	0.0027 (10)
N22	0.0188 (11)	0.0193 (10)	0.0178 (10)	0.0019 (8)	0.0073 (9)	0.0001 (8)
C22	0.0316 (17)	0.0259 (14)	0.0161 (12)	0.0036 (12)	0.0078 (11)	−0.0004 (10)
C23	0.0154 (12)	0.0173 (11)	0.0177 (11)	0.0000 (9)	0.0057 (9)	−0.0020 (9)
N23A	0.0141 (10)	0.0177 (10)	0.0180 (10)	−0.0017 (8)	0.0053 (8)	−0.0026 (8)
C24	0.0194 (13)	0.0213 (12)	0.0200 (12)	−0.0013 (10)	0.0069 (10)	−0.0046 (10)
C25	0.0246 (15)	0.0264 (14)	0.0192 (12)	−0.0077 (11)	0.0064 (11)	−0.0060 (10)
C26	0.0186 (13)	0.0294 (15)	0.0189 (12)	−0.0072 (11)	0.0013 (10)	0.0004 (10)
C27	0.0154 (12)	0.0249 (13)	0.0197 (12)	−0.0015 (10)	0.0029 (10)	0.0023 (10)
C27A	0.0132 (11)	0.0220 (12)	0.0202 (12)	0.0010 (9)	0.0068 (9)	0.0006 (9)
C231	0.0140 (12)	0.0176 (11)	0.0237 (13)	−0.0009 (9)	0.0036 (10)	−0.0003 (9)
N232	0.0184 (12)	0.0170 (10)	0.0341 (14)	−0.0004 (9)	0.0090 (10)	−0.0024 (9)
C233	0.0184 (14)	0.0199 (13)	0.047 (2)	0.0026 (11)	0.0096 (14)	−0.0016 (13)
C234	0.0216 (15)	0.0194 (14)	0.049 (2)	0.0040 (11)	0.0014 (14)	0.0050 (13)
C235	0.0272 (17)	0.0252 (15)	0.0294 (16)	0.0014 (12)	−0.0027 (13)	0.0074 (12)
C236	0.0206 (14)	0.0202 (12)	0.0249 (14)	0.0012 (10)	0.0020 (11)	0.0018 (10)
C31	0.0172 (13)	0.0238 (13)	0.0211 (12)	0.0010 (10)	0.0080 (10)	0.0000 (10)
N32	0.0192 (12)	0.0226 (11)	0.0187 (10)	−0.0015 (9)	0.0050 (9)	0.0007 (8)
C32	0.0300 (17)	0.0339 (16)	0.0169 (12)	−0.0080 (13)	0.0055 (12)	−0.0022 (11)
C34	0.0227 (14)	0.0246 (13)	0.0234 (13)	−0.0070 (11)	0.0091 (11)	−0.0080 (11)
N33A	0.0134 (10)	0.0185 (10)	0.0197 (10)	−0.0029 (8)	0.0038 (8)	−0.0025 (8)
C33	0.0159 (12)	0.0188 (11)	0.0200 (12)	−0.0024 (9)	0.0040 (10)	0.0009 (9)
C35	0.0275 (16)	0.0329 (16)	0.0203 (13)	−0.0129 (13)	0.0077 (12)	−0.0073 (11)
C36	0.0225 (15)	0.0346 (17)	0.0198 (13)	−0.0109 (12)	0.0018 (11)	0.0032 (11)
C37	0.0142 (12)	0.0309 (15)	0.0214 (13)	−0.0030 (11)	0.0030 (10)	0.0048 (11)
C37A	0.0129 (11)	0.0223 (12)	0.0201 (12)	−0.0008 (9)	0.0050 (9)	0.0012 (9)
C331	0.0166 (13)	0.0177 (12)	0.0317 (15)	−0.0025 (10)	0.0005 (11)	0.0018 (11)
N332	0.0189 (13)	0.0178 (11)	0.0460 (17)	−0.0003 (9)	0.0070 (12)	−0.0020 (11)
C333	0.0227 (17)	0.0189 (14)	0.067 (3)	0.0014 (12)	0.0069 (17)	−0.0040 (16)
C334	0.028 (2)	0.0224 (16)	0.073 (3)	0.0022 (14)	−0.006 (2)	0.0070 (18)
C335	0.038 (2)	0.0285 (18)	0.049 (2)	−0.0054 (16)	−0.0103 (19)	0.0149 (17)
C336	0.0292 (18)	0.0340 (18)	0.0308 (17)	−0.0034 (14)	−0.0043 (14)	0.0080 (14)
C41	0.0178 (12)	0.0167 (11)	0.0155 (10)	−0.0004 (9)	0.0054 (9)	−0.0003 (8)
N42	0.0146 (10)	0.0190 (10)	0.0121 (9)	−0.0030 (8)	0.0035 (7)	−0.0016 (7)
C42	0.0250 (15)	0.0262 (14)	0.0151 (11)	0.0037 (11)	0.0005 (10)	0.0007 (10)
C43	0.0139 (11)	0.0171 (11)	0.0149 (10)	−0.0012 (8)	0.0048 (9)	0.0004 (8)
N43A	0.0139 (10)	0.0189 (10)	0.0140 (9)	−0.0005 (8)	0.0052 (8)	0.0003 (7)
C44	0.0209 (13)	0.0224 (12)	0.0149 (11)	0.0006 (10)	0.0075 (10)	0.0006 (9)
C45	0.0237 (14)	0.0289 (14)	0.0140 (11)	0.0027 (11)	0.0046 (10)	0.0028 (10)
C46	0.0267 (16)	0.0265 (14)	0.0188 (12)	0.0082 (12)	0.0055 (11)	0.0036 (10)
C47A	0.0184 (12)	0.0176 (11)	0.0150 (10)	0.0004 (9)	0.0063 (9)	−0.0018 (8)
C47	0.0222 (14)	0.0243 (13)	0.0180 (12)	0.0059 (11)	0.0046 (10)	0.0032 (10)
C431	0.0147 (11)	0.0158 (10)	0.0186 (11)	−0.0008 (8)	0.0064 (9)	0.0021 (8)
N432	0.0180 (11)	0.0177 (10)	0.0212 (11)	−0.0027 (8)	0.0095 (9)	−0.0013 (8)
C433	0.0256 (15)	0.0195 (12)	0.0251 (13)	−0.0032 (10)	0.0144 (12)	−0.0022 (10)
C434	0.0262 (15)	0.0191 (12)	0.0324 (15)	0.0045 (11)	0.0166 (13)	0.0055 (11)
C435	0.0193 (14)	0.0256 (14)	0.0300 (15)	0.0045 (11)	0.0107 (12)	0.0078 (11)
C436	0.0145 (12)	0.0219 (12)	0.0232 (13)	0.0003 (9)	0.0071 (10)	0.0027 (10)

### Bis[2-methyl-3-(pyridin-2-yl)imidazo[1,5-a]pyridin-2-ium] tetraiodocadmate (I) Geometric parameters (Å, º)

**Table:**

Cd1—I14	2.7573 (3)	C234—H234	0.9500
Cd1—I11	2.7764 (3)	C235—C236	1.390 (5)
Cd1—I13	2.7949 (3)	C235—H235	0.9500
Cd1—I12	2.8023 (3)	C236—H236	0.9500
Cd2—I24	2.7575 (3)	C31—N32	1.361 (4)
Cd2—I21	2.7610 (3)	C31—C37A	1.379 (4)
Cd2—I22	2.7943 (3)	C31—H31	0.9500
Cd2—I23	2.7958 (3)	N32—C33	1.357 (4)
C11—N12	1.358 (4)	N32—C32	1.477 (4)
C11—C17A	1.362 (4)	C32—H32A	0.9800
C11—H11	0.9500	C32—H32B	0.9800
N12—C13	1.351 (3)	C32—H32C	0.9800
N12—C12	1.466 (4)	C34—C35	1.353 (5)
C12—H12A	0.9800	C34—N33A	1.394 (4)
C12—H12B	0.9800	C34—H34	0.9500
C12—H12C	0.9800	N33A—C33	1.365 (4)
C13—N13A	1.355 (4)	N33A—C37A	1.389 (4)
C13—C131	1.466 (4)	C33—C331	1.463 (4)
N13A—C17A	1.401 (4)	C35—C36	1.424 (5)
N13A—C14	1.402 (4)	C35—H35	0.9500
C14—C15	1.348 (4)	C36—C37	1.344 (5)
C14—H14	0.9500	C36—H36	0.9500
C15—C16	1.430 (4)	C37—C37A	1.419 (4)
C15—H15	0.9500	C37—H37	0.9500
C16—C17	1.360 (4)	C331—N332	1.345 (5)
C16—H16	0.9500	C331—C336	1.386 (5)
C17—C17A	1.420 (4)	N332—C333	1.334 (5)
C17—H17	0.9500	C333—C334	1.371 (7)
C131—N132	1.349 (4)	C333—H333	0.9500
C131—C136	1.393 (4)	C334—C335	1.371 (8)
N132—C133	1.335 (4)	C334—H334	0.9500
C133—C134	1.383 (5)	C335—C336	1.397 (6)
C133—H133	0.9500	C335—H335	0.9500
C134—C135	1.391 (5)	C336—H336	0.9500
C134—H134	0.9500	C41—N42	1.358 (4)
C135—C136	1.396 (4)	C41—C47A	1.369 (4)
C135—H135	0.9500	C41—H41	0.9500
C136—H136	0.9500	N42—C43	1.350 (3)
C21—N22	1.362 (4)	N42—C42	1.466 (4)
C21—C27A	1.383 (4)	C42—H42A	0.9800
C21—H21	0.9500	C42—H42B	0.9800
N22—C23	1.352 (4)	C42—H42C	0.9800
N22—C22	1.473 (4)	C43—N43A	1.355 (4)
C22—H22A	0.9800	C43—C431	1.462 (4)
C22—H22B	0.9800	N43A—C44	1.399 (4)
C22—H22C	0.9800	N43A—C47A	1.409 (4)
C23—N23A	1.365 (4)	C44—C45	1.354 (4)
C23—C231	1.461 (4)	C44—H44	0.9500
N23A—C24	1.392 (4)	C45—C46	1.429 (4)
N23A—C27A	1.393 (4)	C45—H45	0.9500
C24—C25	1.354 (4)	C46—C47	1.354 (4)
C24—H24	0.9500	C46—H46	0.9500
C25—C26	1.419 (5)	C47A—C47	1.410 (4)
C25—H25	0.9500	C47—H47	0.9500
C26—C27	1.357 (5)	C431—N432	1.344 (4)
C26—H26	0.9500	C431—C436	1.391 (4)
C27—C27A	1.416 (4)	N432—C433	1.340 (4)
C27—H27	0.9500	C433—C434	1.387 (5)
C231—N232	1.346 (4)	C433—H433	0.9500
C231—C236	1.390 (4)	C434—C435	1.394 (5)
N232—C233	1.338 (4)	C434—H434	0.9500
C233—C234	1.386 (6)	C435—C436	1.389 (4)
C233—H233	0.9500	C435—H435	0.9500
C234—C235	1.379 (6)	C436—H436	0.9500
			
I14—Cd1—I11	107.058 (9)	C234—C235—C236	118.8 (3)
I14—Cd1—I13	115.503 (10)	C234—C235—H235	120.6
I11—Cd1—I13	103.726 (9)	C236—C235—H235	120.6
I14—Cd1—I12	102.186 (8)	C235—C236—C231	118.7 (3)
I11—Cd1—I12	117.300 (9)	C235—C236—H236	120.6
I13—Cd1—I12	111.499 (9)	C231—C236—H236	120.6
I24—Cd2—I21	105.915 (9)	N32—C31—C37A	107.2 (3)
I24—Cd2—I22	105.260 (8)	N32—C31—H31	126.4
I21—Cd2—I22	116.026 (10)	C37A—C31—H31	126.4
I24—Cd2—I23	112.854 (9)	C33—N32—C31	110.8 (3)
I21—Cd2—I23	108.090 (9)	C33—N32—C32	127.0 (3)
I22—Cd2—I23	108.791 (9)	C31—N32—C32	122.0 (3)
N12—C11—C17A	107.5 (2)	N32—C32—H32A	109.5
N12—C11—H11	126.3	N32—C32—H32B	109.5
C17A—C11—H11	126.3	H32A—C32—H32B	109.5
C13—N12—C11	110.9 (2)	N32—C32—H32C	109.5
C13—N12—C12	126.6 (2)	H32A—C32—H32C	109.5
C11—N12—C12	122.5 (2)	H32B—C32—H32C	109.5
N12—C12—H12A	109.5	C35—C34—N33A	118.2 (3)
N12—C12—H12B	109.5	C35—C34—H34	120.9
H12A—C12—H12B	109.5	N33A—C34—H34	120.9
N12—C12—H12C	109.5	C33—N33A—C37A	109.6 (2)
H12A—C12—H12C	109.5	C33—N33A—C34	129.6 (3)
H12B—C12—H12C	109.5	C37A—N33A—C34	120.7 (3)
N12—C13—N13A	106.1 (2)	N32—C33—N33A	105.9 (3)
N12—C13—C131	127.4 (3)	N32—C33—C331	128.2 (3)
N13A—C13—C131	126.5 (2)	N33A—C33—C331	125.9 (3)
C13—N13A—C17A	109.3 (2)	C34—C35—C36	122.0 (3)
C13—N13A—C14	129.9 (2)	C34—C35—H35	119.0
C17A—N13A—C14	120.7 (2)	C36—C35—H35	119.0
C15—C14—N13A	118.7 (3)	C37—C36—C35	120.0 (3)
C15—C14—H14	120.6	C37—C36—H36	120.0
N13A—C14—H14	120.6	C35—C36—H36	120.0
C14—C15—C16	121.7 (3)	C36—C37—C37A	119.0 (3)
C14—C15—H15	119.2	C36—C37—H37	120.5
C16—C15—H15	119.2	C37A—C37—H37	120.5
C17—C16—C15	120.2 (3)	C31—C37A—N33A	106.4 (3)
C17—C16—H16	119.9	C31—C37A—C37	133.6 (3)
C15—C16—H16	119.9	N33A—C37A—C37	120.0 (3)
C16—C17—C17A	118.9 (3)	N332—C331—C336	122.8 (3)
C16—C17—H17	120.5	N332—C331—C33	114.9 (3)
C17A—C17—H17	120.5	C336—C331—C33	122.3 (3)
C11—C17A—N13A	106.2 (2)	C333—N332—C331	117.4 (4)
C11—C17A—C17	134.0 (3)	N332—C333—C334	124.0 (4)
N13A—C17A—C17	119.7 (2)	N332—C333—H333	118.0
N132—C131—C136	123.7 (3)	C334—C333—H333	118.0
N132—C131—C13	115.0 (3)	C333—C334—C335	118.5 (4)
C136—C131—C13	121.3 (3)	C333—C334—H334	120.8
C133—N132—C131	116.9 (3)	C335—C334—H334	120.8
N132—C133—C134	124.0 (3)	C334—C335—C336	119.3 (4)
N132—C133—H133	118.0	C334—C335—H335	120.3
C134—C133—H133	118.0	C336—C335—H335	120.3
C133—C134—C135	118.6 (3)	C331—C336—C335	118.0 (4)
C133—C134—H134	120.7	C331—C336—H336	121.0
C135—C134—H134	120.7	C335—C336—H336	121.0
C134—C135—C136	118.8 (3)	N42—C41—C47A	107.7 (2)
C134—C135—H135	120.6	N42—C41—H41	126.1
C136—C135—H135	120.6	C47A—C41—H41	126.1
C131—C136—C135	118.0 (3)	C43—N42—C41	110.9 (2)
C131—C136—H136	121.0	C43—N42—C42	126.4 (2)
C135—C136—H136	121.0	C41—N42—C42	122.6 (2)
N22—C21—C27A	107.1 (2)	N42—C42—H42A	109.5
N22—C21—H21	126.5	N42—C42—H42B	109.5
C27A—C21—H21	126.5	H42A—C42—H42B	109.5
C23—N22—C21	111.0 (2)	N42—C42—H42C	109.5
C23—N22—C22	126.6 (3)	H42A—C42—H42C	109.5
C21—N22—C22	122.0 (3)	H42B—C42—H42C	109.5
N22—C22—H22A	109.5	N42—C43—N43A	106.3 (2)
N22—C22—H22B	109.5	N42—C43—C431	127.4 (3)
H22A—C22—H22B	109.5	N43A—C43—C431	126.4 (2)
N22—C22—H22C	109.5	C43—N43A—C44	130.0 (2)
H22A—C22—H22C	109.5	C43—N43A—C47A	109.4 (2)
H22B—C22—H22C	109.5	C44—N43A—C47A	120.6 (2)
N22—C23—N23A	106.1 (2)	C45—C44—N43A	118.6 (3)
N22—C23—C231	127.3 (3)	C45—C44—H44	120.7
N23A—C23—C231	126.5 (2)	N43A—C44—H44	120.7
C23—N23A—C24	129.5 (3)	C44—C45—C46	121.4 (3)
C23—N23A—C27A	109.6 (2)	C44—C45—H45	119.3
C24—N23A—C27A	120.9 (3)	C46—C45—H45	119.3
C25—C24—N23A	118.2 (3)	C47—C46—C45	120.4 (3)
C25—C24—H24	120.9	C47—C46—H46	119.8
N23A—C24—H24	120.9	C45—C46—H46	119.8
C24—C25—C26	121.9 (3)	C41—C47A—N43A	105.7 (2)
C24—C25—H25	119.1	C41—C47A—C47	134.5 (3)
C26—C25—H25	119.1	N43A—C47A—C47	119.7 (2)
C27—C26—C25	120.4 (3)	C46—C47—C47A	119.1 (3)
C27—C26—H26	119.8	C46—C47—H47	120.4
C25—C26—H26	119.8	C47A—C47—H47	120.4
C26—C27—C27A	118.4 (3)	N432—C431—C436	123.5 (3)
C26—C27—H27	120.8	N432—C431—C43	115.0 (2)
C27A—C27—H27	120.8	C436—C431—C43	121.4 (3)
C21—C27A—N23A	106.2 (2)	C433—N432—C431	117.2 (3)
C21—C27A—C27	133.6 (3)	N432—C433—C434	123.4 (3)
N23A—C27A—C27	120.2 (3)	N432—C433—H433	118.3
N232—C231—C236	123.0 (3)	C434—C433—H433	118.3
N232—C231—C23	115.1 (3)	C433—C434—C435	118.6 (3)
C236—C231—C23	121.8 (3)	C433—C434—H434	120.7
C233—N232—C231	116.8 (3)	C435—C434—H434	120.7
N232—C233—C234	124.1 (3)	C436—C435—C434	118.8 (3)
N232—C233—H233	118.0	C436—C435—H435	120.6
C234—C233—H233	118.0	C434—C435—H435	120.6
C235—C234—C233	118.4 (3)	C435—C436—C431	118.3 (3)
C235—C234—H234	120.8	C435—C436—H436	120.8
C233—C234—H234	120.8	C431—C436—H436	120.8
			
C17A—C11—N12—C13	−0.8 (3)	C37A—C31—N32—C33	1.1 (3)
C17A—C11—N12—C12	177.1 (3)	C37A—C31—N32—C32	−174.4 (3)
C11—N12—C13—N13A	1.5 (3)	C35—C34—N33A—C33	−179.8 (3)
C12—N12—C13—N13A	−176.3 (3)	C35—C34—N33A—C37A	0.4 (4)
C11—N12—C13—C131	−177.1 (3)	C31—N32—C33—N33A	−2.1 (3)
C12—N12—C13—C131	5.1 (5)	C32—N32—C33—N33A	173.1 (3)
N12—C13—N13A—C17A	−1.6 (3)	C31—N32—C33—C331	176.6 (3)
C131—C13—N13A—C17A	177.1 (3)	C32—N32—C33—C331	−8.1 (5)
N12—C13—N13A—C14	179.0 (3)	C37A—N33A—C33—N32	2.3 (3)
C131—C13—N13A—C14	−2.3 (5)	C34—N33A—C33—N32	−177.6 (3)
C13—N13A—C14—C15	−179.7 (3)	C37A—N33A—C33—C331	−176.5 (3)
C17A—N13A—C14—C15	1.1 (4)	C34—N33A—C33—C331	3.6 (5)
N13A—C14—C15—C16	0.4 (5)	N33A—C34—C35—C36	0.9 (5)
C14—C15—C16—C17	−0.9 (5)	C34—C35—C36—C37	−0.8 (5)
C15—C16—C17—C17A	−0.1 (5)	C35—C36—C37—C37A	−0.6 (5)
N12—C11—C17A—N13A	−0.2 (3)	N32—C31—C37A—N33A	0.3 (3)
N12—C11—C17A—C17	−177.1 (3)	N32—C31—C37A—C37	−179.7 (3)
C13—N13A—C17A—C11	1.2 (3)	C33—N33A—C37A—C31	−1.6 (3)
C14—N13A—C17A—C11	−179.4 (3)	C34—N33A—C37A—C31	178.2 (3)
C13—N13A—C17A—C17	178.6 (3)	C33—N33A—C37A—C37	178.4 (3)
C14—N13A—C17A—C17	−2.0 (4)	C34—N33A—C37A—C37	−1.8 (4)
C16—C17—C17A—C11	178.0 (3)	C36—C37—C37A—C31	−178.1 (3)
C16—C17—C17A—N13A	1.5 (5)	C36—C37—C37A—N33A	1.9 (4)
N12—C13—C131—N132	−142.3 (3)	N32—C33—C331—N332	155.3 (3)
N13A—C13—C131—N132	39.3 (4)	N33A—C33—C331—N332	−26.2 (4)
N12—C13—C131—C136	39.7 (4)	N32—C33—C331—C336	−26.9 (5)
N13A—C13—C131—C136	−138.7 (3)	N33A—C33—C331—C336	151.7 (3)
C136—C131—N132—C133	−1.3 (4)	C336—C331—N332—C333	1.9 (5)
C13—C131—N132—C133	−179.3 (3)	C33—C331—N332—C333	179.8 (3)
C131—N132—C133—C134	−1.7 (4)	C331—N332—C333—C334	1.3 (5)
N132—C133—C134—C135	2.9 (5)	N332—C333—C334—C335	−2.7 (6)
C133—C134—C135—C136	−1.0 (4)	C333—C334—C335—C336	0.9 (6)
N132—C131—C136—C135	3.1 (4)	N332—C331—C336—C335	−3.6 (5)
C13—C131—C136—C135	−179.1 (3)	C33—C331—C336—C335	178.8 (3)
C134—C135—C136—C131	−1.8 (4)	C334—C335—C336—C331	2.0 (6)
C27A—C21—N22—C23	−0.8 (3)	C47A—C41—N42—C43	1.3 (3)
C27A—C21—N22—C22	172.5 (3)	C47A—C41—N42—C42	−175.5 (3)
C21—N22—C23—N23A	2.1 (3)	C41—N42—C43—N43A	−1.6 (3)
C22—N22—C23—N23A	−170.8 (3)	C42—N42—C43—N43A	175.0 (3)
C21—N22—C23—C231	−174.1 (3)	C41—N42—C43—C431	177.7 (3)
C22—N22—C23—C231	13.0 (5)	C42—N42—C43—C431	−5.6 (5)
N22—C23—N23A—C24	176.9 (3)	N42—C43—N43A—C44	−179.7 (3)
C231—C23—N23A—C24	−6.8 (5)	C431—C43—N43A—C44	1.0 (5)
N22—C23—N23A—C27A	−2.7 (3)	N42—C43—N43A—C47A	1.3 (3)
C231—C23—N23A—C27A	173.6 (3)	C431—C43—N43A—C47A	−178.1 (3)
C23—N23A—C24—C25	−179.3 (3)	C43—N43A—C44—C45	179.6 (3)
C27A—N23A—C24—C25	0.2 (4)	C47A—N43A—C44—C45	−1.5 (4)
N23A—C24—C25—C26	−1.0 (5)	N43A—C44—C45—C46	−0.3 (5)
C24—C25—C26—C27	0.6 (5)	C44—C45—C46—C47	1.7 (5)
C25—C26—C27—C27A	0.7 (5)	N42—C41—C47A—N43A	−0.5 (3)
N22—C21—C27A—N23A	−0.9 (3)	N42—C41—C47A—C47	177.5 (3)
N22—C21—C27A—C27	−179.0 (3)	C43—N43A—C47A—C41	−0.5 (3)
C23—N23A—C27A—C21	2.2 (3)	C44—N43A—C47A—C41	−179.6 (3)
C24—N23A—C27A—C21	−177.4 (3)	C43—N43A—C47A—C47	−178.9 (3)
C23—N23A—C27A—C27	−179.3 (3)	C44—N43A—C47A—C47	2.0 (4)
C24—N23A—C27A—C27	1.0 (4)	C45—C46—C47—C47A	−1.2 (5)
C26—C27—C27A—C21	176.4 (3)	C41—C47A—C47—C46	−178.4 (3)
C26—C27—C27A—N23A	−1.5 (4)	N43A—C47A—C47—C46	−0.6 (5)
N22—C23—C231—N232	−153.9 (3)	N42—C43—C431—N432	143.8 (3)
N23A—C23—C231—N232	30.6 (4)	N43A—C43—C431—N432	−37.0 (4)
N22—C23—C231—C236	28.6 (5)	N42—C43—C431—C436	−38.5 (4)
N23A—C23—C231—C236	−146.9 (3)	N43A—C43—C431—C436	140.7 (3)
C236—C231—N232—C233	−1.9 (5)	C436—C431—N432—C433	2.4 (4)
C23—C231—N232—C233	−179.3 (3)	C43—C431—N432—C433	−179.9 (2)
C231—N232—C233—C234	−1.4 (5)	C431—N432—C433—C434	1.3 (4)
N232—C233—C234—C235	2.8 (6)	N432—C433—C434—C435	−2.9 (5)
C233—C234—C235—C236	−0.9 (5)	C433—C434—C435—C436	0.7 (4)
C234—C235—C236—C231	−2.1 (5)	C434—C435—C436—C431	2.7 (4)
N232—C231—C236—C235	3.6 (5)	N432—C431—C436—C435	−4.5 (4)
C23—C231—C236—C235	−179.1 (3)	C43—C431—C436—C435	178.0 (3)

### Bis[2-methyl-3-(pyridin-2-yl)imidazo[1,5-a]pyridin-2-ium] tetraiodocadmate (I) Hydrogen-bond geometry (Å, º)

**Table:**

*D*—H···*A*	*D*—H	H···*A*	*D*···*A*	*D*—H···*A*
C11—H11···I11	0.95	3.25	3.972 (3)	134
C11—H11···I12	0.95	3.31	3.918 (3)	124
C12—H12*A*···I24^i^	0.98	3.00	3.929 (3)	158
C12—H12*B*···I24^ii^	0.98	2.93	3.853 (3)	158
C12—H12*C*···I12	0.98	3.31	4.198 (3)	152
C14—H14···N132	0.95	2.50	3.020 (4)	114
C15—H15···I22^iii^	0.95	3.07	3.956 (3)	156
C17—H17···I11	0.95	3.20	3.963 (3)	139
C22—H22*B*···I22^iii^	0.98	3.12	4.062 (4)	162
C24—H24···I13	0.95	3.15	3.877 (3)	135
C24—H24···N232	0.95	2.35	2.916 (4)	118
C25—H25···I12	0.95	3.00	3.796 (3)	142
C27—H27···I24^i^	0.95	3.03	3.796 (3)	139
C234—H234···I12^iv^	0.95	3.20	4.144 (3)	171
C31—H31···I21^v^	0.95	3.22	3.934 (3)	134
C32—H32*A*···I13	0.98	3.31	3.962 (4)	126
C32—H32*B*···I12^vi^	0.98	3.24	4.209 (3)	170
C34—H34···I23	0.95	3.17	3.991 (3)	146
C34—H34···N332	0.95	2.30	2.870 (5)	118
C35—H35···I22	0.95	3.01	3.875 (3)	152
C37—H37···I14^iv^	0.95	3.20	3.916 (3)	134
C334—H334···I22^i^	0.95	3.14	4.028 (4)	157
C41—H41···I22	0.95	3.20	3.897 (3)	132
C42—H42*A*···I14^iv^	0.98	3.00	3.953 (3)	165
C42—H42*C*···I14^vii^	0.98	2.94	3.826 (3)	151
C44—H44···N432	0.95	2.48	2.994 (4)	114
C45—H45···I12^vi^	0.95	3.10	3.997 (3)	158
C47—H47···I21	0.95	3.19	3.945 (3)	137

Symmetry codes: (i) *x*−1/2, −*y*+3/2, *z*−1/2; (ii) −*x*+1/2, *y*−1/2, −*z*+1/2; (iii) −*x*+1, −*y*+1, −*z*+1; (iv) *x*+1/2, −*y*+1/2, *z*+1/2; (v) −*x*+3/2, *y*−1/2, −*z*+1/2; (vi) −*x*+1, −*y*+1, −*z*; (vii) −*x*+3/2, *y*+1/2, −*z*+1/2.

### Bis[2-methyl-3-(pyridin-2-yl)imidazo[1,5-a]pyridin-2-ium] 1.5-chloride 0.5-nitrate trihydrate (II) Crystal data

**Table:**

2C_13_H_12_N_3_^+^·1.5Cl^−^·0.5NO_3_^−^·3H_2_O	*Z* = 2
*M**_r_* = 558.74	*F*(000) = 586
Triclinic, *P*1	*D*_x_ = 1.398 Mg m^−^^3^
Hall symbol: -P 1	Cu *K*α radiation, λ = 1.54178 Å
*a* = 7.3959 (5) Å	Cell parameters from 2491 reflections
*b* = 10.2889 (8) Å	θ = 2.4–66.6°
*c* = 18.5155 (10) Å	µ = 2.14 mm^−^^1^
α = 88.208 (5)°	*T* = 100 K
β = 95.033 (5)°	Needle, light brown
γ = 108.916 (5)°	0.23 × 0.05 × 0.03 mm
*V* = 1327.71 (16) Å^3^	

### Bis[2-methyl-3-(pyridin-2-yl)imidazo[1,5-a]pyridin-2-ium] 1.5-chloride 0.5-nitrate trihydrate (II) Data collection

**Table:**

Oxford Diffraction Gemini diffractometer	4693 independent reflections
Radiation source: sealed X-ray tube	3366 reflections with *I* > 2σ(*I*)
Mirror monochromator	*R*_int_ = 0.050
Detector resolution: 10.4738 pixels mm^-1^	θ_max_ = 67.3°, θ_min_ = 2.4°
ω scans	*h* = −8→8
Absorption correction: analytical (CrysAlis Pro; Rigaku OD, 2016)	*k* = −11→12
*T*_min_ = 0.777, *T*_max_ = 0.942	*l* = −18→22
11325 measured reflections	

### Bis[2-methyl-3-(pyridin-2-yl)imidazo[1,5-a]pyridin-2-ium] 1.5-chloride 0.5-nitrate trihydrate (II) Refinement

**Table:**

Refinement on *F*^2^	9 restraints
Least-squares matrix: full	Hydrogen site location: mixed
*R*[*F*^2^ > 2σ(*F*^2^)] = 0.047	H atoms treated by a mixture of independent and constrained refinement
*wR*(*F*^2^) = 0.121	*w* = 1/[σ^2^(*F*_o_^2^) + (0.0483*P*)^2^ + 0.4628*P*] where *P* = (*F*_o_^2^ + 2*F*_c_^2^)/3
*S* = 1.02	(Δ/σ)_max_ = 0.045
4693 reflections	Δρ_max_ = 0.26 e Å^−^^3^
385 parameters	Δρ_min_ = −0.26 e Å^−^^3^

### Bis[2-methyl-3-(pyridin-2-yl)imidazo[1,5-a]pyridin-2-ium] 1.5-chloride 0.5-nitrate trihydrate (II) Special details

**Table:**

Geometry. All esds (except the esd in the dihedral angle between two l.s. planes) are estimated using the full covariance matrix. The cell esds are taken into account individually in the estimation of esds in distances, angles and torsion angles; correlations between esds in cell parameters are only used when they are defined by crystal symmetry. An approximate (isotropic) treatment of cell esds is used for estimating esds involving l.s. planes.
Refinement. One anions site was modelled as being disordered between a Cl- and a NO3- ion with site occupancies constrained to 0.5 after trial refinement. Water molecule hydrogen geometries were restrained to ideal values.

### Bis[2-methyl-3-(pyridin-2-yl)imidazo[1,5-a]pyridin-2-ium] 1.5-chloride 0.5-nitrate trihydrate (II) Fractional atomic coordinates and isotropic or equivalent isotropic displacement parameters (Å^2^)

**Table:**

	*x*	*y*	*z*	*U*_iso_*/*U*_eq_	Occ. (<1)
C11	0.5212 (4)	−0.0797 (3)	0.20815 (15)	0.0450 (6)	
H11	0.4386	−0.1648	0.1888	0.054*	
N12	0.5524 (3)	−0.0437 (2)	0.27913 (12)	0.0448 (5)	
C12	0.4527 (4)	−0.1317 (3)	0.33735 (16)	0.0557 (8)	
H12A	0.5304	−0.1868	0.3583	0.084*	
H12B	0.3281	−0.1928	0.3174	0.084*	
H12C	0.4333	−0.074	0.3751	0.084*	
C13	0.6785 (4)	0.0847 (3)	0.28750 (15)	0.0436 (6)	
N13A	0.7256 (3)	0.1306 (2)	0.22022 (11)	0.0392 (5)	
C14	0.8549 (4)	0.2565 (3)	0.19923 (15)	0.0419 (6)	
H14	0.9218	0.3248	0.2341	0.05*	
C15	0.8809 (4)	0.2772 (3)	0.12871 (16)	0.0464 (7)	
H15	0.9685	0.3617	0.1138	0.056*	
C16	0.7814 (4)	0.1768 (3)	0.07516 (16)	0.0512 (7)	
H16	0.801	0.1958	0.0254	0.061*	
C17	0.6587 (4)	0.0544 (3)	0.09528 (16)	0.0484 (7)	
H17	0.593	−0.0131	0.0599	0.058*	
C17A	0.6293 (4)	0.0281 (3)	0.16953 (15)	0.0420 (6)	
C131	0.7484 (4)	0.1624 (3)	0.35480 (15)	0.0470 (7)	
N132	0.7557 (3)	0.2948 (2)	0.35039 (12)	0.0470 (6)	
C133	0.8101 (4)	0.3688 (3)	0.41137 (16)	0.0548 (8)	
H133	0.8169	0.4626	0.4095	0.066*	
C134	0.8572 (5)	0.3143 (4)	0.47729 (17)	0.0650 (9)	
H134	0.8908	0.3694	0.5197	0.078*	
C135	0.8545 (5)	0.1806 (4)	0.48040 (17)	0.0683 (10)	
H135	0.8885	0.1423	0.5248	0.082*	
C136	0.8011 (4)	0.1009 (3)	0.41742 (16)	0.0589 (9)	
H136	0.8009	0.0085	0.4174	0.071*	
C21	0.3963 (4)	0.2931 (3)	0.13167 (14)	0.0391 (6)	
H21	0.4506	0.339	0.0895	0.047*	
N22	0.4269 (3)	0.3465 (2)	0.19957 (11)	0.0401 (5)	
C22	0.5590 (4)	0.4855 (3)	0.21763 (15)	0.0478 (7)	
H22A	0.4865	0.5502	0.2163	0.072*	
H22B	0.6557	0.5138	0.1823	0.072*	
H22C	0.6224	0.4847	0.2663	0.072*	
C23	0.3290 (4)	0.2537 (3)	0.24656 (14)	0.0386 (6)	
N23A	0.2338 (3)	0.1378 (2)	0.20761 (11)	0.0374 (5)	
C24	0.1083 (4)	0.0130 (3)	0.23010 (14)	0.0402 (6)	
H24	0.0817	−0.0021	0.2795	0.048*	
C25	0.0260 (4)	−0.0853 (3)	0.18033 (15)	0.0431 (6)	
H25	−0.0607	−0.1705	0.1948	0.052*	
C26	0.0666 (4)	−0.0642 (3)	0.10589 (15)	0.0441 (6)	
H26	0.0076	−0.1358	0.0719	0.053*	
C27	0.1869 (4)	0.0557 (3)	0.08345 (14)	0.0421 (6)	
H27	0.2134	0.0692	0.034	0.051*	
C27A	0.2738 (4)	0.1618 (3)	0.13487 (13)	0.0372 (6)	
C231	0.3247 (4)	0.2691 (3)	0.32528 (14)	0.0446 (7)	
N232	0.3284 (3)	0.1595 (3)	0.36524 (12)	0.0492 (6)	
C233	0.3338 (5)	0.1723 (4)	0.43704 (16)	0.0624 (9)	
H233	0.3361	0.0952	0.4663	0.075*	
C234	0.3363 (5)	0.2894 (4)	0.47135 (18)	0.0710 (10)	
H234	0.3448	0.2942	0.5228	0.085*	
C235	0.3262 (5)	0.3989 (4)	0.42941 (18)	0.0675 (10)	
H235	0.3236	0.4804	0.4514	0.081*	
C236	0.3198 (4)	0.3902 (3)	0.35404 (16)	0.0565 (8)	
H236	0.3123	0.4648	0.3237	0.068*	
Cl1	−0.13460 (14)	0.74736 (10)	0.35172 (5)	0.0385 (2)	0.5
N1	−0.13460 (14)	0.74736 (10)	0.35172 (5)	0.0385 (2)	0.5
O11	−0.1116 (7)	0.8679 (5)	0.3658 (3)	0.0704 (13)	0.5
O12	−0.0043 (8)	0.7052 (6)	0.3432 (3)	0.0879 (16)	0.5
O13	−0.2989 (7)	0.6577 (5)	0.3587 (3)	0.0818 (15)	0.5
Cl2	0.19972 (10)	0.62995 (7)	0.10394 (4)	0.0532 (2)	
O1	0.7669 (3)	0.5341 (2)	0.03747 (13)	0.0547 (5)	
O2	0.6162 (3)	0.7445 (2)	0.04753 (13)	0.0558 (5)	
O3	0.0954 (4)	0.5668 (4)	0.26639 (16)	0.0982 (11)	
H1AO	0.881 (3)	0.563 (3)	0.0552 (16)	0.067 (11)*	
H1BO	0.767 (5)	0.496 (4)	−0.0016 (14)	0.103 (16)*	
H2AO	0.664 (4)	0.682 (3)	0.0471 (18)	0.069 (11)*	
H2BO	0.513 (3)	0.715 (3)	0.0667 (19)	0.080 (13)*	
H3AO	−0.017 (3)	0.552 (5)	0.269 (2)	0.121*	
H3BO	0.126 (6)	0.596 (5)	0.2244 (15)	0.121*	

### Bis[2-methyl-3-(pyridin-2-yl)imidazo[1,5-a]pyridin-2-ium] 1.5-chloride 0.5-nitrate trihydrate (II) Atomic displacement parameters (Å^2^)

**Table:**

	*U*^11^	*U*^22^	*U*^33^	*U*^12^	*U*^13^	*U*^23^
C11	0.0369 (15)	0.0351 (14)	0.0588 (17)	0.0093 (12)	−0.0104 (12)	−0.0033 (12)
N12	0.0332 (12)	0.0357 (12)	0.0563 (14)	0.0025 (10)	−0.0089 (10)	0.0080 (10)
C12	0.0436 (17)	0.0422 (16)	0.0625 (18)	−0.0073 (13)	−0.0086 (13)	0.0131 (13)
C13	0.0305 (14)	0.0387 (15)	0.0528 (16)	0.0023 (11)	−0.0054 (11)	0.0087 (12)
N13A	0.0293 (12)	0.0361 (12)	0.0497 (13)	0.0085 (9)	−0.0014 (9)	0.0045 (9)
C14	0.0311 (14)	0.0365 (14)	0.0555 (17)	0.0082 (11)	0.0022 (11)	0.0064 (12)
C15	0.0408 (16)	0.0412 (15)	0.0607 (18)	0.0170 (13)	0.0112 (13)	0.0071 (13)
C16	0.0566 (19)	0.0489 (17)	0.0542 (17)	0.0237 (15)	0.0116 (14)	0.0021 (13)
C17	0.0490 (18)	0.0429 (16)	0.0565 (17)	0.0193 (14)	0.0015 (13)	−0.0045 (13)
C17A	0.0355 (15)	0.0356 (14)	0.0553 (16)	0.0140 (12)	−0.0034 (12)	−0.0006 (12)
C131	0.0305 (14)	0.0436 (16)	0.0515 (16)	−0.0066 (12)	−0.0043 (11)	0.0076 (12)
N132	0.0339 (13)	0.0430 (13)	0.0502 (13)	−0.0054 (10)	−0.0002 (10)	0.0025 (10)
C133	0.0407 (17)	0.0513 (17)	0.0552 (18)	−0.0082 (13)	0.0020 (13)	0.0007 (14)
C134	0.054 (2)	0.067 (2)	0.0474 (17)	−0.0162 (16)	−0.0027 (14)	0.0007 (15)
C135	0.055 (2)	0.064 (2)	0.0541 (18)	−0.0188 (16)	−0.0165 (15)	0.0160 (15)
C136	0.0449 (18)	0.0501 (18)	0.0590 (19)	−0.0106 (14)	−0.0117 (14)	0.0134 (14)
C21	0.0388 (15)	0.0398 (14)	0.0381 (14)	0.0136 (12)	−0.0016 (11)	0.0028 (11)
N22	0.0364 (12)	0.0379 (12)	0.0426 (12)	0.0092 (10)	−0.0026 (9)	0.0009 (9)
C22	0.0457 (17)	0.0372 (15)	0.0512 (16)	0.0025 (12)	−0.0030 (12)	0.0011 (12)
C23	0.0288 (14)	0.0402 (14)	0.0438 (14)	0.0083 (11)	−0.0015 (10)	−0.0004 (11)
N23A	0.0298 (11)	0.0376 (12)	0.0435 (12)	0.0100 (9)	−0.0004 (9)	0.0005 (9)
C24	0.0279 (13)	0.0434 (15)	0.0465 (15)	0.0084 (11)	0.0010 (11)	0.0037 (12)
C25	0.0307 (14)	0.0419 (15)	0.0520 (16)	0.0070 (12)	−0.0039 (11)	0.0009 (12)
C26	0.0385 (16)	0.0403 (15)	0.0507 (16)	0.0114 (12)	−0.0081 (12)	−0.0052 (12)
C27	0.0407 (15)	0.0427 (15)	0.0433 (14)	0.0158 (12)	−0.0044 (11)	−0.0009 (11)
C27A	0.0345 (14)	0.0403 (14)	0.0373 (13)	0.0143 (11)	−0.0015 (10)	0.0025 (11)
C231	0.0294 (14)	0.0518 (17)	0.0455 (15)	0.0024 (12)	0.0038 (11)	−0.0070 (13)
N232	0.0371 (13)	0.0538 (15)	0.0419 (13)	−0.0057 (11)	0.0050 (10)	0.0063 (11)
C233	0.0517 (19)	0.070 (2)	0.0449 (17)	−0.0101 (16)	0.0090 (13)	0.0057 (15)
C234	0.067 (2)	0.077 (2)	0.0469 (18)	−0.0102 (18)	0.0198 (16)	−0.0031 (18)
C235	0.062 (2)	0.069 (2)	0.061 (2)	0.0015 (17)	0.0141 (16)	−0.0214 (18)
C236	0.0487 (19)	0.0581 (19)	0.0564 (18)	0.0075 (15)	0.0081 (14)	−0.0055 (15)
Cl1	0.0372 (6)	0.0403 (6)	0.0307 (5)	0.0024 (4)	0.0031 (4)	−0.0018 (4)
N1	0.0372 (6)	0.0403 (6)	0.0307 (5)	0.0024 (4)	0.0031 (4)	−0.0018 (4)
O11	0.069 (3)	0.053 (3)	0.079 (3)	0.004 (2)	0.013 (2)	0.003 (2)
O12	0.062 (3)	0.087 (4)	0.112 (4)	0.018 (3)	0.013 (3)	−0.023 (3)
O13	0.069 (3)	0.067 (3)	0.094 (4)	−0.002 (3)	0.014 (3)	−0.018 (3)
Cl2	0.0423 (4)	0.0422 (4)	0.0674 (5)	0.0008 (3)	0.0099 (3)	−0.0093 (3)
O1	0.0464 (14)	0.0543 (13)	0.0655 (14)	0.0190 (11)	0.0043 (10)	−0.0054 (11)
O2	0.0440 (13)	0.0359 (11)	0.0848 (15)	0.0068 (10)	0.0161 (11)	0.0035 (10)
O3	0.0689 (18)	0.100 (2)	0.0867 (19)	−0.0167 (17)	−0.0188 (14)	0.0375 (16)

### Bis[2-methyl-3-(pyridin-2-yl)imidazo[1,5-a]pyridin-2-ium] 1.5-chloride 0.5-nitrate trihydrate (II) Geometric parameters (Å, º)

**Table:**

C11—N12	1.356 (4)	C22—H22A	0.98
C11—C17A	1.363 (4)	C22—H22B	0.98
C11—H11	0.95	C22—H22C	0.98
N12—C13	1.352 (3)	C23—N23A	1.363 (3)
N12—C12	1.473 (4)	C23—C231	1.475 (4)
C12—H12A	0.98	N23A—C24	1.395 (3)
C12—H12B	0.98	N23A—C27A	1.401 (3)
C12—H12C	0.98	C24—C25	1.343 (4)
C13—N13A	1.353 (3)	C24—H24	0.95
C13—C131	1.463 (4)	C25—C26	1.431 (4)
N13A—C17A	1.399 (3)	C25—H25	0.95
N13A—C14	1.404 (3)	C26—C27	1.345 (4)
C14—C15	1.337 (4)	C26—H26	0.95
C14—H14	0.95	C27—C27A	1.419 (4)
C15—C16	1.426 (4)	C27—H27	0.95
C15—H15	0.95	C231—N232	1.335 (4)
C16—C17	1.356 (4)	C231—C236	1.383 (4)
C16—H16	0.95	N232—C233	1.336 (4)
C17—C17A	1.415 (4)	C233—C234	1.374 (5)
C17—H17	0.95	C233—H233	0.95
C131—N132	1.346 (4)	C234—C235	1.366 (5)
C131—C136	1.390 (4)	C234—H234	0.95
N132—C133	1.339 (4)	C235—C236	1.397 (4)
C133—C134	1.391 (5)	C235—H235	0.95
C133—H133	0.95	C236—H236	0.95
C134—C135	1.370 (5)	Cl1—O12	1.201 (6)
C134—H134	0.95	Cl1—O11	1.229 (5)
C135—C136	1.399 (5)	Cl1—O13	1.279 (5)
C135—H135	0.95	O1—H1AO	0.836 (18)
C136—H136	0.95	O1—H1BO	0.832 (18)
C21—N22	1.359 (3)	O2—H2AO	0.832 (17)
C21—C27A	1.364 (4)	O2—H2BO	0.832 (18)
C21—H21	0.95	O3—H3AO	0.802 (19)
N22—C23	1.343 (3)	O3—H3BO	0.848 (18)
N22—C22	1.475 (3)		
			
N12—C11—C17A	107.7 (2)	C23—N22—C21	110.6 (2)
N12—C11—H11	126.1	C23—N22—C22	126.1 (2)
C17A—C11—H11	126.1	C21—N22—C22	123.1 (2)
C13—N12—C11	110.5 (2)	N22—C22—H22A	109.5
C13—N12—C12	125.8 (2)	N22—C22—H22B	109.5
C11—N12—C12	123.6 (2)	H22A—C22—H22B	109.5
N12—C12—H12A	109.5	N22—C22—H22C	109.5
N12—C12—H12B	109.5	H22A—C22—H22C	109.5
H12A—C12—H12B	109.5	H22B—C22—H22C	109.5
N12—C12—H12C	109.5	N22—C23—N23A	106.5 (2)
H12A—C12—H12C	109.5	N22—C23—C231	128.1 (2)
H12B—C12—H12C	109.5	N23A—C23—C231	125.4 (2)
N12—C13—N13A	106.3 (2)	C23—N23A—C24	129.7 (2)
N12—C13—C131	127.9 (2)	C23—N23A—C27A	108.9 (2)
N13A—C13—C131	125.8 (2)	C24—N23A—C27A	121.4 (2)
C13—N13A—C17A	109.4 (2)	C25—C24—N23A	118.5 (2)
C13—N13A—C14	129.1 (2)	C25—C24—H24	120.8
C17A—N13A—C14	121.5 (2)	N23A—C24—H24	120.8
C15—C14—N13A	118.0 (3)	C24—C25—C26	121.2 (3)
C15—C14—H14	121	C24—C25—H25	119.4
N13A—C14—H14	121	C26—C25—H25	119.4
C14—C15—C16	122.2 (3)	C27—C26—C25	120.9 (2)
C14—C15—H15	118.9	C27—C26—H26	119.6
C16—C15—H15	118.9	C25—C26—H26	119.6
C17—C16—C15	120.0 (3)	C26—C27—C27A	118.9 (2)
C17—C16—H16	120	C26—C27—H27	120.5
C15—C16—H16	120	C27A—C27—H27	120.5
C16—C17—C17A	119.4 (3)	C21—C27A—N23A	106.1 (2)
C16—C17—H17	120.3	C21—C27A—C27	134.8 (2)
C17A—C17—H17	120.3	N23A—C27A—C27	119.1 (2)
C11—C17A—N13A	106.0 (2)	N232—C231—C236	123.7 (3)
C11—C17A—C17	135.1 (3)	N232—C231—C23	115.1 (2)
N13A—C17A—C17	118.9 (2)	C236—C231—C23	121.1 (3)
N132—C131—C136	124.1 (3)	C231—N232—C233	116.7 (3)
N132—C131—C13	114.7 (2)	N232—C233—C234	124.3 (3)
C136—C131—C13	121.2 (3)	N232—C233—H233	117.8
C133—N132—C131	117.1 (2)	C234—C233—H233	117.8
N132—C133—C134	122.9 (3)	C235—C234—C233	118.1 (3)
N132—C133—H133	118.6	C235—C234—H234	121
C134—C133—H133	118.6	C233—C234—H234	121
C135—C134—C133	119.3 (3)	C234—C235—C236	119.6 (3)
C135—C134—H134	120.3	C234—C235—H235	120.2
C133—C134—H134	120.3	C236—C235—H235	120.2
C134—C135—C136	119.2 (3)	C231—C236—C235	117.5 (3)
C134—C135—H135	120.4	C231—C236—H236	121.2
C136—C135—H135	120.4	C235—C236—H236	121.2
C131—C136—C135	117.3 (3)	O12—Cl1—O11	123.2 (4)
C131—C136—H136	121.3	O12—Cl1—O13	117.1 (4)
C135—C136—H136	121.3	O11—Cl1—O13	118.6 (3)
N22—C21—C27A	107.8 (2)	H1AO—O1—H1BO	107 (3)
N22—C21—H21	126.1	H2AO—O2—H2BO	109 (2)
C27A—C21—H21	126.1	H3AO—O3—H3BO	111 (3)

### Bis[2-methyl-3-(pyridin-2-yl)imidazo[1,5-a]pyridin-2-ium] 1.5-chloride 0.5-nitrate trihydrate (II) Hydrogen-bond geometry (Å, º)

**Table:**

*D*—H···*A*	*D*—H	H···*A*	*D*···*A*	*D*—H···*A*
O1—H1*AO*···Cl2^i^	0.836 (18)	2.339 (18)	3.174 (2)	176 (3)
O1—H1*BO*···Cl2^ii^	0.832 (18)	2.41 (2)	3.229 (2)	171 (4)
O2—H2*AO*···O1	0.832 (17)	1.925 (18)	2.755 (3)	175 (4)
O2—H2*BO*···Cl2	0.832 (18)	2.352 (18)	3.178 (2)	172 (4)
O3—H3*AO*···Cl1	0.802 (19)	2.95 (5)	3.398 (4)	118 (4)
O3—H3*BO*···Cl2	0.848 (18)	2.33 (2)	3.166 (3)	168 (5)
O3—H3*AO*···O12	0.802 (19)	2.10 (5)	2.363 (7)	99 (4)
C11—H11···Cl2^iii^	0.95	2.71	3.640 (3)	166
C12—H12*A*···Cl1^iv^	0.98	2.79	3.638 (4)	146
C14—H14···N132	0.95	2.53	3.024 (4)	112
C14—H14···O3^i^	0.95	2.47	3.330 (4)	151
C15—H15···Cl2^i^	0.95	2.75	3.671 (3)	165
C17—H17···O2^iii^	0.95	2.57	3.244 (3)	128
C24—H24···N232	0.95	2.51	3.019 (4)	114
C27—H27···O2^ii^	0.95	2.48	3.255 (3)	139
C236—H236···O3	0.95	2.35	3.160 (5)	143

Symmetry codes: (i) *x*+1, *y*, *z*; (ii) −*x*+1, −*y*+1, −*z*; (iii) *x*, *y*−1, *z*; (iv) *x*+1, *y*−1, *z*.
