# Supplementary figures and images for: Crystal structures of an imidazo[1,5-a]pyridinium-based ligand and its (C13H12N3)2[CdI4] hybrid salt
Source: Acta Crystallogr E Crystallogr Commun. 2019 Jul 19;75(Pt 8):1209–14. doi: 10.1107/S2056989019009964 (PMC6690455; doi:10.1107/S2056989019009964)

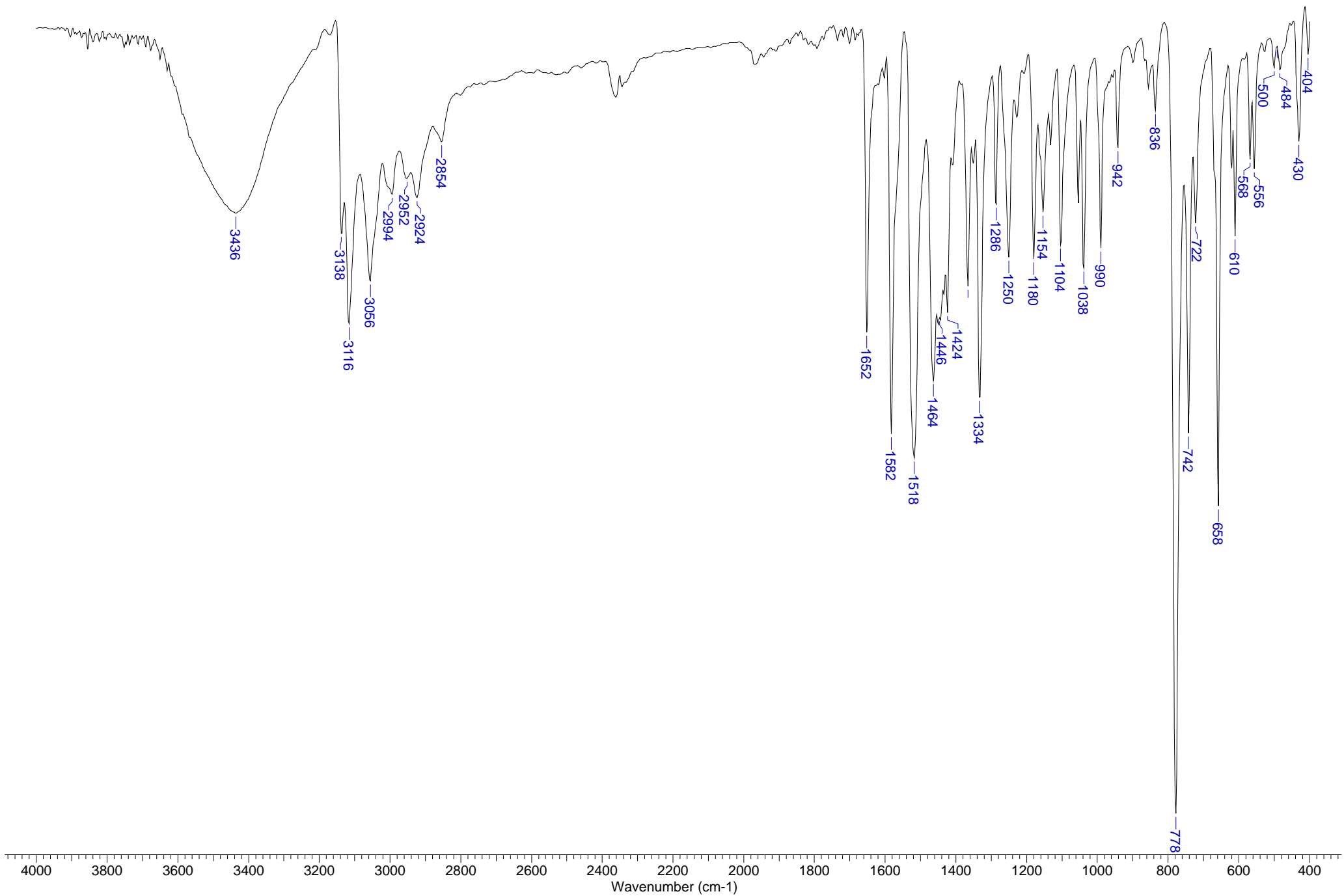

Supplement: Supplementary file 4 [file e-75-01209-sup4.pdf]

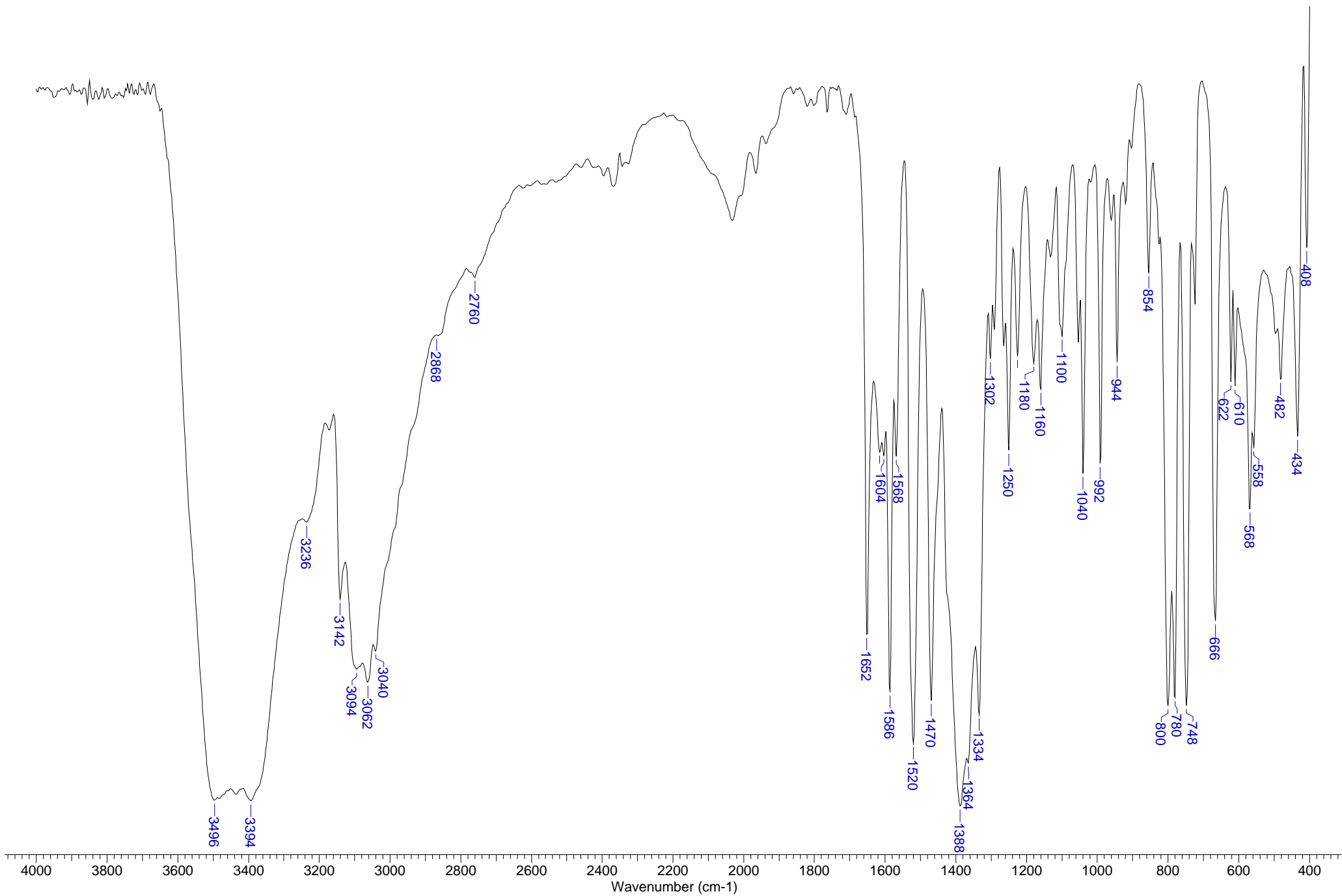

Supplement: Supplementary file 5 [file e-75-01209-sup5.pdf]
